# Climate extremes, land–climate feedbacks and land-use forcing at 1.5°C

**DOI:** 10.1098/rsta.2016.0450

**Published:** 2018-04-02

**Authors:** Sonia I. Seneviratne, Richard Wartenburger, Benoit P. Guillod, Annette L. Hirsch, Martha M. Vogel, Victor Brovkin, Detlef P. van Vuuren, Nathalie Schaller, Lena Boysen, Katherine V. Calvin, Jonathan Doelman, Peter Greve, Petr Havlik, Florian Humpenöder, Tamas Krisztin, Daniel Mitchell, Alexander Popp, Keywan Riahi, Joeri Rogelj, Carl-Friedrich Schleussner, Jana Sillmann, Elke Stehfest

**Affiliations:** 1 Institute for Atmospheric and Climate Science, ETH Zurich, 8092 Zurich, Switzerland; 2 Institute for Environmental Decisions, ETH Zurich, 8092 Zurich, Switzerland; 3 Max-Planck Institute for Meteorology, Bundesstrasse 53, 20146 Hamburg, Germany; 4 PBL Netherlands Environmental Assessment Agency, PO Box 303, Bilthoven 3720 AH, The Netherlands; 5 Copernicus Institute, Utrecht University, Heidelberglaan 2, 3584 CS Utrecht, The Netherlands; 6 CICERO, P.O. Box 1129, Blindern, 0318 Oslo, Norway; 7 Pacific Northwest National Laboratory (PNNL), Joint Global Change Research Institute, College Park, MD 20740, USA; 8 International Institute for Applied Systems Analysis (IIASA), Laxenburg 2361, Austria; 9 Potsdam Institute for Climate Impact Research (PIK), Member of the Leibniz Association, PO Box 60 12 03, 14412 Potsdam, Germany; 10 School of Geographical Sciences, University Road, Clifton, Bristol BS8 1SS, UK; 11 Climate Analytics, Ritterstrasse 3, 10969 Berlin, Germany

**Keywords:** climate extremes, 1.5°C scenarios, land-use changes, regional climate change, climate projections, land–climate interactions

## Abstract

This article investigates projected changes in temperature and water cycle extremes at 1.5°C of global warming, and highlights the role of land processes and land-use changes (LUCs) for these projections. We provide new comparisons of changes in climate at 1.5°C versus 2°C based on empirical sampling analyses of transient simulations versus simulations from the ‘Half a degree Additional warming, Prognosis and Projected Impacts’ (HAPPI) multi-model experiment. The two approaches yield similar overall results regarding changes in climate extremes on land, and reveal a substantial difference in the occurrence of regional extremes at 1.5°C versus 2°C. Land processes mediated through soil moisture feedbacks and land-use forcing play a major role for projected changes in extremes at 1.5°C in most mid-latitude regions, including densely populated areas in North America, Europe and Asia. This has important implications for low-emissions scenarios derived from integrated assessment models (IAMs), which include major LUCs in ambitious mitigation pathways (e.g. associated with increased bioenergy use), but are also shown to differ in the simulated LUC patterns. Biogeophysical effects from LUCs are not considered in the development of IAM scenarios, but play an important role for projected regional changes in climate extremes, and are thus of high relevance for sustainable development pathways.

This article is part of the theme issue ‘The Paris Agreement: understanding the physical and social challenges for a warming world of 1.5°C above pre-industrial levels'.

## 1. Introduction

The 2015 Paris Agreement aims to hold the average global warming compared to pre-industrial levels to ‘well below 2°C’, and ‘to pursue efforts’ to limit it to 1.5°C above pre-industrial levels [1]. These targets refer to changes in global mean temperature. Their implications for regional impacts remain in part uncertain, mostly because past assessment reports of the Intergovernmental Panel on Climate Change (IPCC) generally focused on high-emissions rather than low-emissions scenarios [2]. For instance, of the four emissions pathways considered within the Working Group I Contribution to the IPCC 5th Assessment Report (AR5), only one ‘representative climate pathway’ (RCP2.6) included a relatively high probability of climate stabilization below 2°C by 2100 [2,3].

**Figure 1.**
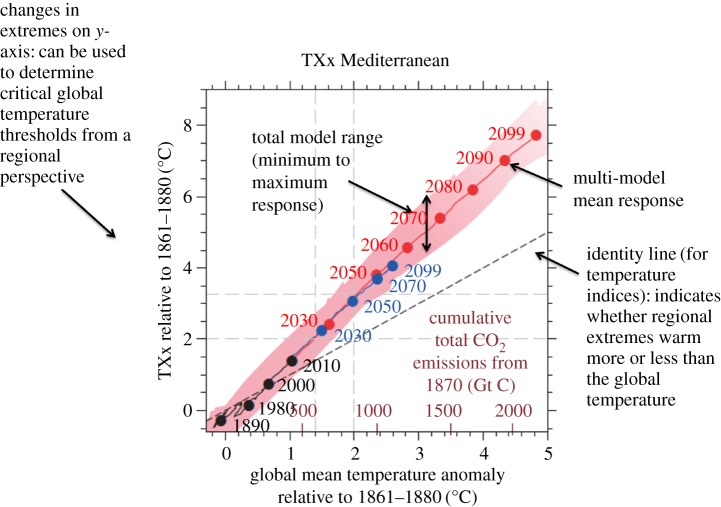
Empirical scaling relationship (ESR) between changes in yearly maximum daily midday temperature and global temperature warming in the Mediterranean region based on simulations from the fifth phase of the Coupled Model Intercomparison Project (CMIP5), including explanatory annotations. The red line indicates the multi-model mean of the RCP8.5 CMIP5 simulations, and the blue line indicates the multi-model mean of the RCP4.5 CMIP5 simulations (from ref. [8], adapted from ref. [5]).

As a consequence, and to provide assessments of changes in climate at 1.5°C and 2°C of global warming, recent assessments have used alternative approaches to classical Coupled Modelling Intercomparison Project (CMIP) [4] experiments to assess changes in regional climate, extremes and impacts at those warming levels [5–7]. An approach that has been used extensively consists of an empirical sampling analysis, whereby regional patterns in relevant variables are assessed in available climate model simulations at given global warming levels over an averaging time window [5–8]. When using gradual sampling over different levels of global warming, this approach can be used to derive an empirical scaling relationship (hereafter referred to as ESR) between a given quantity of interest (e.g. changes in an extreme index in a given region) and global mean temperature warming [5,8] (see figure 1 and §2). Analyses have shown that derived ESRs are often linear for multi-model averages of a range of extreme indices in the ensemble simulations of the fifth phase of the CMIP experiment (CMIP5) and mostly independent of the considered emissions scenarios [5,8] (see, however, §2 for some exceptions). In the case of regional temperature means and extremes (on land), the changes are found to be generally much larger than for the global mean temperature, in some cases with up to 1.5 to 3 times larger warming [5]. This behaviour is due to several mechanisms, among others to land–atmosphere interactions, as highlighted hereafter.

Despite their attractive simplicity and consistency, empirical sampling analyses based on transient simulations may not be representative of projections with a full climate stabilization at lower levels of warming. Hence, while they have received substantial attention in the recent literature [5,8], it is important to evaluate their level of consistency with other more in-depth procedures relying on actual modelling of climate conditions at 1.5°C or 2°C of global warming. This is particularly relevant in the context of the upcoming IPCC special report on 1.5°C of warming (hereafter referred to as IPCC SR15), which will provide for the first time an assessment of changes in climate impacts at 1.5°C of global warming, in comparison with 2°C and higher levels of warming (http://ipcc.ch/report/sr15/).

To evaluate changes at 1.5°C and 2°C of global warming in conditions closer to climate equilibrium (i.e. when the sea surface temperatures are fixed to conditions associated with these respective warming levels), as well as to allow for high sampling of internal climate variability, a new model intercomparison experiment called ‘Half a degree Additional warming, Prognosis and Projected Impacts’ (HAPPI) was initiated [9]. The HAPPI experimental set-up consists of time slice experiments with forced sea surface temperatures (SSTs) corresponding either to the present day or to future conditions at 1.5°C or 2°C of global warming, derived from CMIP5 simulations associated with RCP2.6 and (for 2°C only) RCP4.5 scenarios [9]. We will evaluate the level of consistency between the ESR approach and the HAPPI experiment for the derivation of changes in temperature and precipitation extremes at different levels of global warming.

Another main issue addressed in the present article is the role of land–climate interactions and land-use changes for low-emissions scenarios, with a focus on changes in temperature extremes. As demonstrated hereafter, recent publications have shown that, in several land regions, the warming in temperature extremes is much higher than the corresponding warming in the global mean temperature [5,8,10]. A main mechanism underlying this feature in mid-latitude regions is related to soil moisture–temperature feedbacks, namely additional warming incurred by lack of evaporative cooling in regions projected to be affected by increased drying [11–14]. This implies that some of the regional response of temperature extremes, which may be assessed as a ‘regional transient climate response’ (TCRreg), can be more strongly affected by regional processes than by the global mean temperature transient climate response (generally referred to as TCR, hereafter expressed as TCRglob).

As a consequence of the role of regional processes in affecting TCRreg, biogeophysical land-use forcing, e.g. through irrigation or changes in land surface albedo associated with changes in land cover or land management, is found to be a key driver of changes in regional climate extremes, especially in low-emissions scenarios [15,16]. This is of key relevance for the development of climate change scenarios, since land-use changes (e.g. associated with changes in agricultural production, reforestation/afforestation, or the cultivation of bioenergy crops, in some case with carbon capture and storage (BECCS)) are a major component of integrated assessment models (IAMs) that underlie the derivation of these scenarios [3,17]. However, these changes in land use are only considered from the perspective of carbon cycle impacts in IAM models, and do not encompass the associated biogeophysical feedbacks (see also [16]). As highlighted in this article, the integration of these biogeophysical feedbacks in future IAM developments would be critical to explore the full dimension of low-emissions scenarios. We illustrate this need by displaying the projected change in land use associated with well-established IAM models for newly developed low-emissions scenarios.

The article is structured as follows: §2 describes the methods and data used in this article. Then, §3 compares projected changes in regional extremes at 1.5°C versus 2°C using the ESR approach with results from the HAPPI simulations, and §4 identifies in which regions significant differences between changes in climate extremes can be found at 1.5°C versus 2°C of global warming in the two sets of estimates. Section 5 provides more background on the recent literature highlighting the role of land–climate interactions and land forcing on regional projections in low-emissions scenarios, and §6 provides an overview of changes in land use in newly developed low-emissions scenarios from state-of-the-art IAM models. Finally, §7 provides a summary of the highlighted results and the main conclusions of this article.

## 2. Methods and data

### (a) Assessing changes in regional climate extremes for different levels of global temperature warming: the empirical scaling relationship

The ESR approach consists of relating changes in regional climate variables to the change in the global mean temperature, by using an empirical sampling based on transient climate simulations (figure 1). More background on this approach can be found in references [5] and [8]. The ESR approach is related to the ‘time sampling approach’ described in James *et al*. [7], except that instead of focusing on single time slices, it derives a relationship of the response for a range of global temperature levels. This yields an ‘empirical’ pattern scaling. Compared to the traditional pattern scaling literature [7,18,19], it does not rely on any assumption regarding dependence (e.g. linearity).

Nonetheless, as highlighted in the introduction, results have shown a surprisingly high degree of linearity in the regional changes in extreme event intensity at different levels of global warming (from pre-industrial conditions to more than 4°C of global warming) based on multi-model means [5,8]. These results are consistent with analyses suggesting a robust forced response pattern in projections of changes in temperature and precipitation extremes [20]. Interestingly, it was also found that the ESRs for temperature and precipitation extremes in the CMIP5 multi-model ensemble are mostly independent of the considered emissions scenarios [5,8], suggesting little impact of the timing of greenhouse gas emissions in the emissions scenarios considered so far.

These results also suggest that differences in land-use or aerosol forcing in the RCP scenarios used in the CMIP5 simulations overall did not strongly affect their associated ESR behaviour for temperature and precipitation extremes [5,8]. In the case of land-use forcing, this might reflect the lack of diversity in regional forcing options considered in the RCP scenarios, as highlighted hereafter. With respect to aerosols, for regions with very high current aerosol loadings, recent studies suggest that the effects of aerosol mitigation on regional extremes could nonetheless be comparable to the effects of a 0.5°C global temperature difference [21,22]. It should furthermore be noted that mean precipitation projections also exhibit a scenario dependence in the RCP ensemble [23], and that the time dimension can be relevant for variables with large inertia (e.g. sea-level rise [2]). Despite these caveats, the fact that the ESR approach appears to emulate well the regional responses of extremes for different global temperature levels—almost independently of the scenario pathway and time frame within the twenty-first century—makes it attractive for investigating low-emissions scenarios based on existing simulations for higher-emissions pathways.

Because of its ease of interpretation, and the fact that the analyses can be performed based on already existing simulations, the ESR approach has been used in several publications assessing changes in regional climate extremes at 1.5°C versus 2°C, and higher levels of global warming [5,7].

In the present article, we use the CMIP5-based ESR analyses data processed in Wartenburger *et al*. [8], based on slightly modified analyses compared to Seneviratne *et al*. [5] (see [8] for more details and links to the data). The ESR-CMIP5 estimates are based on simulations from 26 Earth system models (ESMs) with interactive ocean including up to 10 ensembles per model. Table 1 provides the list of analysed models. We compare these estimates to results from the HAPPI experiment (see §2b). The analysed climate extreme indices are described in §2c.

**Table 1.** Names of models used in the CMIP5-based ESR estimates (§2a) with associated institutions.

model	hosting institution
ACCESS1-0	Commonwealth Scientific and Industrial Research Organization (CSIRO)/Bureau of Meteorology (BOM), Australia
bcc-csm1-1	Beijing Climate Center, China Meteorological Administration
bcc-csm1-1-m	Beijing Climate Center, China Meteorological Administration
CanESM2	Canadian Centre for Climate Modelling and Analysis
CCSM4	National Center for Atmospheric Research, USA
CMCC-CM	Centro Euro-Mediterraneo per i Cambiamenti Climatici, Italy
CMCC-CMS	Centro Euro-Mediterraneo per i Cambiamenti Climatici, Italy
CNRM-CM5	Centre National de Recherches Météorologiques/Centre Européen de Recherche et Formation Avancées en Calcul Scientifique, France
CSIRO-Mk3-6-0	Commonwealth Scientific and Industrial Research Organization/Queensland Climate Change Centre of Excellence, Australia
FGOALS-s2	LASG, Institute of Atmospheric Physics, Chinese Academy of Sciences
GFDL-CM3	NOAA Geophysical Fluid Dynamics Laboratory, USA
GFDL-ESM2G	NOAA Geophysical Fluid Dynamics Laboratory, USA
GFDL-ESM2M	NOAA Geophysical Fluid Dynamics Laboratory, USA
HadGEM2-CC	Met Office Hadley Centre, United Kingdom
HadGEM2-ES	Met Office Hadley Centre, United Kingdom
inmcm4	Institute for Numerical Mathematics, Russia
IPSL-CM5A-LR	Institut Pierre-Simon Laplace, France
IPSL-CM5A-MR	Institut Pierre-Simon Laplace, France
IPSL-CM5B-LR	Institut Pierre-Simon Laplace, France
MIROC-ESM	Japan Agency for Marine-Earth Science and Technology, Atmosphere and Ocean Research Institute (University of Tokyo)/National Institute for Environmental Studies
MIROC-ESM-CHEM	Japan Agency for Marine-Earth Science and Technology, Atmosphere and Ocean Research Institute (University of Tokyo)/National Institute for Environmental Studies
MIROC5	Japan Agency for Marine-Earth Science and Technology, Atmosphere and Ocean Research Institute (University of Tokyo)/National Institute for Environmental Studies
MPI-ESM-LR	Max Planck Institute for Meteorology, Germany
MPI-ESM-MR	Max Planck Institute for Meteorology, Germany
MRI-CGCM3	Meteorological Research Institute, Japan
NorESM1-M	Norwegian Climate Centre

### (b) Assessing changes in regional climate extremes for different levels of global temperature warming: the HAPPI experiment

The HAPPI protocol [9] consists of coupled land–atmosphere initial condition ensemble simulations with prescribed sea surface temperatures (SSTs), sea-ice, greenhouse gas (GHG) and aerosol concentrations, and solar and volcanic activity, which coincide with three forced climate states. This includes a present-climate decade (2006–2015) that is simulated using observed external forcings (such as observed SSTs, sea ice, GHG and land use), and two future-climate decades (2091–2100) with the global mean temperature forced to 1.5°C or 2°C, using prescribed SST conditions. The 1.5°C future-climate experiment uses the atmospheric concentrations of the year 2095 taken directly from RCP2.6. RCP2.6 is used because coincidentally the multi-model mean of CMIP5 models stabilizes at 1.5°C at the end of the century. For the SSTs and sea ice, the CMIP5 multi-model mean SST/sea-ice anomaly between 2091–2100 and the present day is added to the observed values which were used in the present-climate experiment. Incidentally, this means that multi-model spread in SSTs at this warming level is not accounted for in the HAPPI set-up. The 2°C future-climate experiment employs a similar experimental design, but uses a weighted combination of RCP2.6 and RCP4.5 multi-model mean for the GHGs, SSTs and sea ice, as there is no single RCP scenario that directly stabilizes at 2°C. As aerosols are not well mixed, and should not be averaged between two RCP scenarios, these are kept the same for both the 1.5°C and 2°C future-climate experiments (see [9] for details). Natural forcings (solar and volcanic activity) correspond to the present-climate decade in all climate states. Land use in future-climate decades is the same for both 1.5°C and 2°C and is taken from RCP2.6. Hence, HAPPI implicitly assumes that both climate targets require the same changes in land use.

We note that the HAPPI results reflect changes with respect to present-day conditions and do not include simulations for the pre-industrial time period, while the ESR-CMIP5 estimates (§2a) can be derived for different periods, including pre-industrial conditions (see table 2 for an overview). For this reason, the main comparisons between the ESR-CMIP5 and HAPPI estimates are performed with respect to present-day conditions (see figures in §3). However, to also relate some of the results to the 1.5°C and 2°C global warming targets (which are defined with respect to pre-industrial and not present-day climate), we perform the following conversion. In analyses investigating responses as a function of the overall global mean warming since the pre-industrial period (see figure in §4), we further add the ESR-based differences of the indices between present-day and pre-industrial warming levels, assuming a global mean temperature difference of 1°C, to the HAPPI anomalies defined with respect to the present day.

**Table 2.** Description of definition of pre-industrial, present-day, 1.5°C-climate and 2°C-climate conditions in ESR-CMIP5 and HAPPI analyses.

	ESR-CMIP5 (based on [5] and [8])	HAPPI [9]
pre-industrial conditions	1861–1880	*1861–1880 (used for definition of global warming references, no simulations)*
present-day conditions	Δ*T*_glob_ = 1°C in CMIP5 simulations for twenty-first century	2006–2015 (observed SSTs, sea ice, GHG and land use)
1.5°C-climate	Δ*T*_glob_ = 1.5°C in CMIP5 simulations for twenty-first century	simulations driven with SST and sea ice conditions consistent with Δ*T*_glob_ = 1.5°C in CMIP5 RCP2.6 simulations; land use from the RCP 2.6 scenario
2°C-climate	Δ*T*_glob_ = 2°C in CMIP5 simulations for twenty-first century	simulations driven with SST and sea ice conditions consistent with Δ*T*_glob_ = 2°C in CMIP5 RCP2.6 and RCP4.5 simulations; land use from the RCP2.6 scenario

The HAPPI estimates of changes in extremes for the considered climate conditions are derived from simulations conducted with five models with ensembles in the range of 100–500 members each, but we only consider 100 members per model here for consistency across models. The considered extreme indices (§2c) were calculated for the first 100 ensemble members (#1 to #100) of the following models: CanAM4, CAM4, ECHAM6-3-LR, MIROC5 and NorESM1-happi. For each of the HAPPI models and the two experiments considered (1.5°C relative to pre-industrial and 2°C relative to pre-industrial), we compute regionally averaged differences of the indices (scenario period – reference period, consisting of 10 years of data per ensemble member).

An advantage of the HAPPI simulations compared to ESR-CMIP5 analyses based on transient climate simulations is that they assess climate conditions that are closer to climate equilibrium for the different considered temperature levels. Indeed, in the HAPPI experiments, the SSTs and sea ice are fixed and prescribed based on simulations reaching lower warming levels by the end of the twenty-first century (nonetheless, because of the used set-up, these are obviously not consistent with conditions at climate equilibrium after several millennia). On the other hand, the HAPPI estimates rely on a smaller number of ESMs, and do not account for spread in oceanic conditions. Given these various differences, it is important to assess if analyses based on the HAPPI framework would result in different conclusions with respect to changes in regional climate and extremes at 1.5°C versus 2°C global mean temperature levels when compared with analyses based on the ESR approach applied to transient CMIP5 experiments.

### (c) Analysed extreme indices

In the analyses, we use extreme indices processed using well-established definitions and procedures [24,25]. We focus on four indices, namely the annual maximum daytime temperature (TXx), the annual minimum night-time temperature (TNn), the annual maximum consecutive 5-day precipitation (Rx5day) and the consecutive dry days (CDD). TXx and TNn quantify temperature extremes, Rx5day is a measure for long-lasting heavy precipitation and CDD is a measure of dryness.

### (d) Land-use changes in integrated assessment models

Changes in land use are an essential element of recently derived IAM scenarios [3,26], in particular in preparation for the sixth phase of the CMIP experiment [17]. The considered changes in land use are generally related to changes in agricultural area for food production, as well as to changes related to climate mitigation through biofuel production or reforestation and afforestation. Many deep mitigation scenarios rely strongly on the use of BECCS for negative emissions.

We analyse here changes in land use in simulations from state-of-the art IAMs for low-emissions scenarios. The considered scenarios are derived for the RCP1.9 and RCP2.6 emissions pathways. We further focus here on two major shared socio-economic pathway (SSP) narratives: SSP1 (‘sustainability’) and SSP2 (‘middle of the road’) [27].

We consider four state-of-the-art IAMs: GCAM [28], MESSAGE-GLOBIOM [29], IMAGE [30] and REMIND-MAgPIE [31]. The reader is referred to the respective publications for more background on each model. Land-cover classes are nearly identical among these models and only minor reassignments had to be applied to have joint classes for the comparisons (table 3). For each of the IAMs, we show the instantaneous land-cover state (expressed as land-cover fractions) for a particular (common) year in the future projections after remapping the model output to a common regular 0.5° grid, or differences of these datasets between considered time frames.

**Table 3.** Mapping of model-specific land-cover classes to common land-cover classes (leftmost column) used in this paper. Aggregation of multiple classes is denoted by plus (‘+’) signs.

mapped land-cover class	GCAM (PNNL)	MESSAGE-GLOBIOM (IIASA)	IMAGE (PBL)	REMIND-MAgPIE (PIK)
cropland	corn + fibre crop+ fodder grass+ fodder herb+ miscellaneous crop+ oil crop + other grain+ palm fruit + rice+ root tuber + sugar crop+ wheat	arable land	cropland	16 food/feed crop types (e.g. temperate and tropical cereals, maize, rice, oilseeds, roots), both rain-fed and irrigated systems, and two second-generation bioenergy crop types (grassy and woody)
grassland	grassland	grassland	pasture	grassland
forest	forest	forest	forest	forest
other land and urban				
	shrub + snow +sparse + urban	other land	other land + urban	other natural land (e.g. non-forest natural vegetation, abandoned agricultural land, deserts) + built-up area

While land use was also implicitly included in the RCP set [3,32], the new set of IAM scenarios explicitly separates the role of climate policy and other socio-economic drivers (SSPs), including quantifications of five different socio-economic futures that can be paired with multiple radiative forcing targets. Such a framework enables a more comprehensive view on how land use may evolve and the associated impacts. Moreover, each scenario was quantified by multiple IAMs, unlike the RCPs. As a result, we can examine the uncertainties surrounding the land-use patterns of different combination of SSPs and radiative forcing targets.

## 3. Changes in regional climate extremes at 1.5°C and 2°C of global warming: comparison of ESR- and HAPPI-based estimates

**Figure 2.**
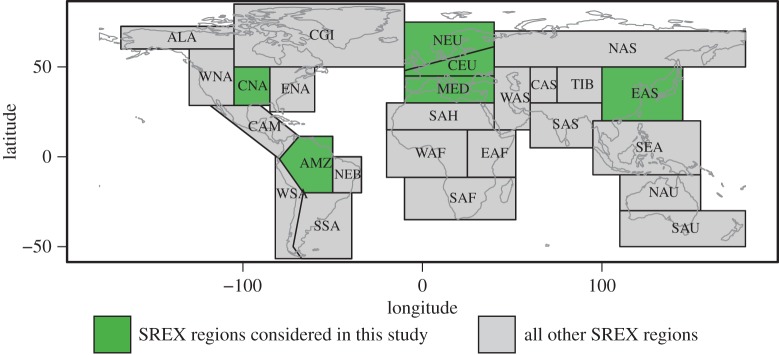
Regions considered in the analyses (from the IPCC SREX report [33]). (Online version in colour.)

**Figure 3.**
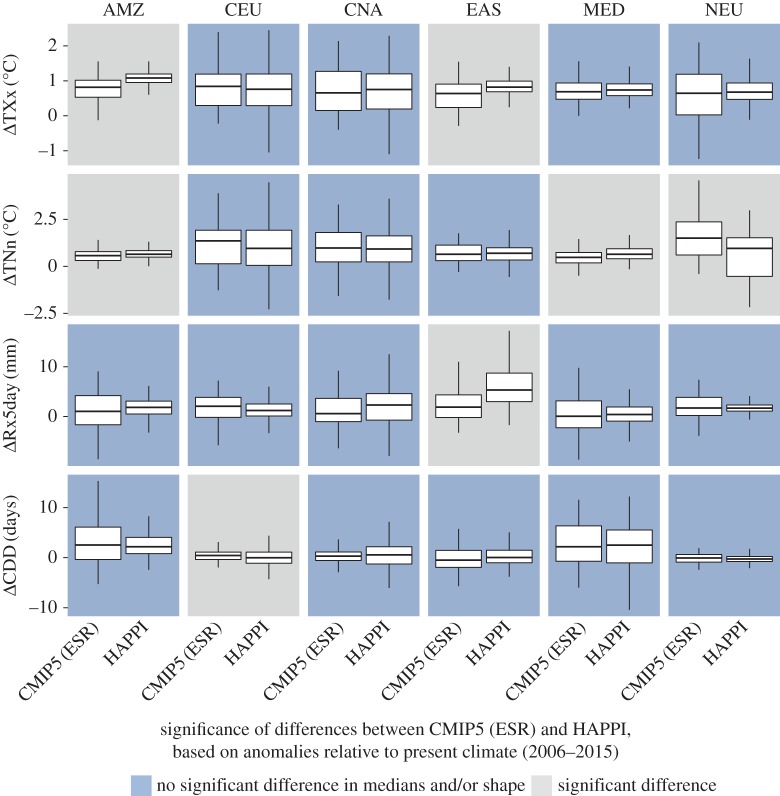
Changes in regional extremes at 1.5°C of global warming estimated from the ESR approach based on CMIP5 simulations (‘CMIP5 (ESR)’) based on [5,8], and from the HAPPI simulations. The changes are computed with respect to present-day climate (1°C of warming for ESR-CMIP5, respectively 2006–2015 in HAPPI). Displayed are box plots indicating the median (middle horizontal line), interquartile range (25th and 75th percentile) indicated with lower (25th percentile) and upper (75th percentile) hinges of the box, and whiskers extending to the lowest (highest) value that is within 1.5 times the interquartile range of the upper (lower) hinge (for figures including outliers, see electronic supplementary material). Analyses are provided for changes in maximum daytime temperature (TXx), minimum night-time temperature (TNn), annual maximum consecutive 5-day precipitation (Rx5day) and consecutive dry days (CDD), and for six regions (figure 2) as defined in the IPCC SREX report [33]: AMZ (Amazon), CEU (Central Europe), CNA (Central North America), EAS (Eastern Asia), MED (Mediterranean) and NEU (Northern Europe). Blue background shading indicates when there is no significant different in the median or the shape between the ESR-CMIP5 and HAPPI estimates (based on Wilcoxon rank-sum test). (Online version in colour.)

**Figure 4.**
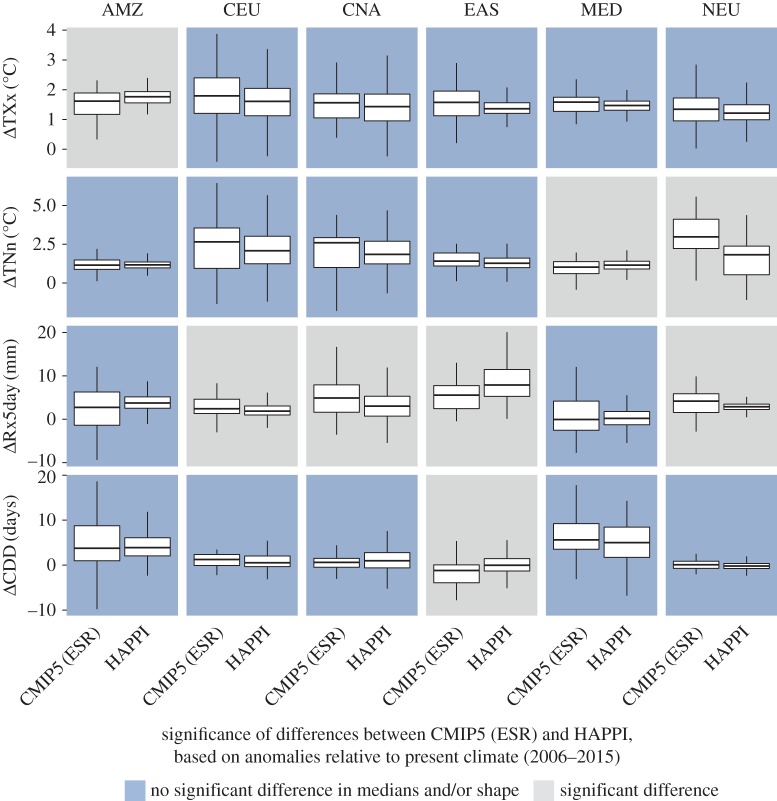
Same as figure 3 but for changes at 2°C of global warming. (Online version in colour.)

In the following, we provide comparisons of changes in regional climate extremes at 1.5°C and 2°C of global warming compared to pre-industrial levels based on the ESR and HAPPI approaches. For the comparison, we focus on six regions (figure 2) which have been shown to display substantial changes between 1.5°C and 2°C of global warming for the considered extremes [5,8], based on domains from the IPCC Special Report on Extremes (IPCC SREX) [33, ch. 3]: the Amazon region (AMZ), Central Europe (CEU), Central North America (CNA), Eastern Asia (EAS), the Mediterranean region (MED) and Northern Europe (NEU). Figures 3 and 4 display changes in four different extreme indices, TXx, TNn, Rx5day and CDD (see §2b and [24,25]), for the two considered methods at 1.5°C and 2°C of global warming, respectively.

**Figure 5.**
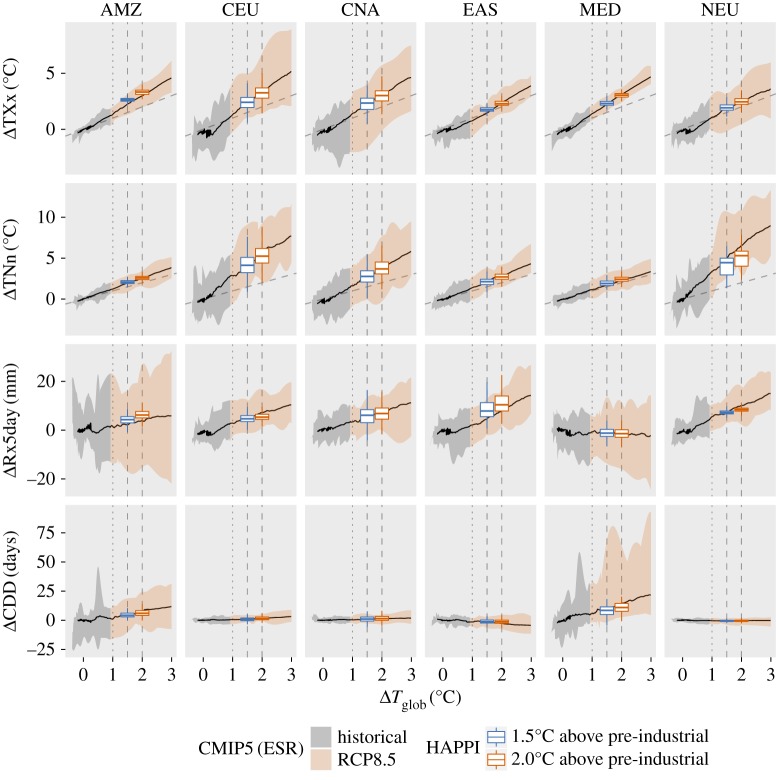
Changes in regional extremes compared to pre-industrial (1861–1880) levels estimated from the ESR approach applied to the CMIP5 ensemble based on historical (grey shading) and RCP8.5 simulations derived from [8], including respective analyses from HAPPI simulations (orange shading), which are superimposed for 1.5°C and 2.0°C of global warming (blue and red box plots). Analyses are provided for changes in maximum daytime temperature (TXx), minimum night-time temperature (TNn), annual maximum consecutive 5-day precipitation (Rx5day) and consecutive dry days (CDD), and for six regions (figure 2) as defined in chapter 3 of the IPCC SREX [33]: AMZ (Amazon), CEU (Central Europe), CNA (Central North America), EAS (Eastern Asia), MED (Mediterranean) and NEU (Northern Europe). The upper and lower hinges of the box plots represent the first and third quartile. The whiskers (coloured in blue or red) extend to the highest (lowest) value that is within 1.5 times the interquartile range of the upper (lower) hinge. The central line of each box plot indicates the median value. The dashed inclined grey line on the TXx and TNn plots indicates the 1 : 1 line of equivalent changes in regional temperature extremes and global temperature. The vertical lines indicate changes at 1°C of global warming (dotted line, corresponding to present-day climate), and 1.5°C and 2°C of global warming (dashed lines).

Despite large differences in the procedures underlying the ESR-CMIP5 estimates and HAPPI simulations, analyses reveal that the results based on these two approaches are overall surprisingly consistent (figures 3 and 4; see electronic supplementary material for results for other regions). A comparison of the median and shape of the distributions by means of a Wilcoxon rank-sum test shows that, in the majority of comparisons (for different indices and regions), the distributions from the ESR approach are not significantly different from the ones of the HAPPI simulations—although there are a few exceptions. This conclusion is also confirmed when displaying the full ESR relationships with superimposed results from the HAPPI simulations (figure 5).

The results are overall not qualitatively different when considering other SREX regions (see electronic supplementary material). Nonetheless, some indices in a few regions display substantial differences (e.g. Rx5day in EAS and CAM, TNn in WAS, TXx in ALA). In addition, there seems to be a slight tendency for the ESR-CMIP5 estimates to display a broader spread (interquartile range) than the HAPPI estimates. This may reflect the fact that the ESR-CMIP5 estimates are based on a larger number of ESMs (§§2a and 2b), and also that the HAPPI set-up does not take into account uncertainty of climate changes in the oceans, since it prescribes a uniform change in SSTs compared to the considered present-day decade.

Hence, we can conclude from these analyses that changes in temperature and water cycle extremes based on the ESR-CMIP5 approach for the considered indices should be generally robust compared to analyses using time slice experiments with forced SSTs consistent with the given investigated levels of global warming, although there are a few exceptions.

## 4. Identifying regions with substantial changes in temperature and water cycle extremes at 1.5°C versus 2°C of global warming

One important question related to the evaluation of changes at 1.5°C of global warming is the extent to which some impacts could be avoided at this level of warming compared to conditions at 2°C of global warming. Hence, it is relevant to assess whether there are significant differences in the occurrence of climate extremes at these two warming levels [6,8].

**Figure 6.**
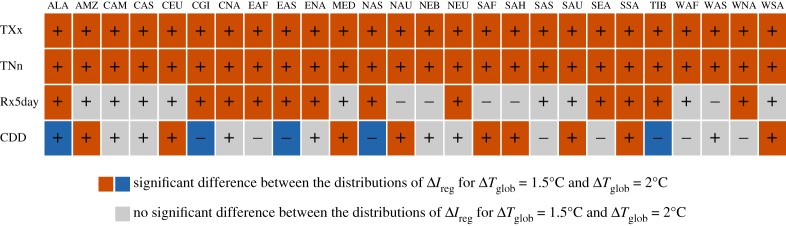
ESR-CMIP5-based assessment of differences of changes in regional climate extremes between 1.5°C and 2°C of global warming: significant increases are shown in red, significant decreases are shown in blue and non-significant changes are shown in grey. See figure 2 for regions' acronyms. (Adapted from [8].)

**Figure 7.**
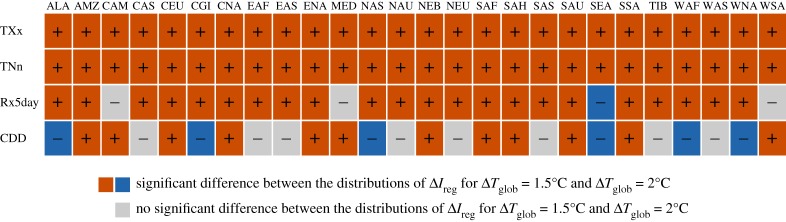
HAPPI-based assessment of differences of changes in regional climate extremes between 1.5°C and 2°C of global warming: significant increases are shown in red, significant decreases are shown in blue and non-significant changes are shown in grey. See figure 2 for regions' acronyms.

Figures 6 and 7 provide analyses similar to those performed in Wartenburger *et al*. [8] displaying whether regional changes in climate extremes at 1.5°C versus 2°C of global warming are significantly different. These analyses are provided for the four climate extreme indices considered in figures 3–5, i.e. TXx, TNn, Rx5day and CDD. As highlighted in Wartenburger *et al*. [8] based on ESR-CMIP5 analyses, differences are generally found to be significant for temperature extremes (for TXx and TNn, in all regions). This is also the case for the results of the HAPPI simulations (figure 7), and also consistent with recent analyses of health-related heat extremes [34]. Results are more inconsistent for water cycle extremes, as also previously highlighted [8]. However, it is interesting to note that the analyses based on the HAPPI simulations seem to show more frequently significant changes between the two warming levels, also for the water cycle extremes. This could be due to the fact that the larger number of ensemble members per model allows for more robust statistics. On the other hand, it is also possible that the HAPPI-based ensemble underestimates inter-model uncertainty due to the smaller number of considered climate models (five) compared to those used in the ESR-CMIP5 estimates (26) and the prescribed SST conditions (see also previous section). This could be in particular the case for the estimates of changes in CDD, which are in part inconsistent (also in terms of sign) between the ESR-CMIP5 and HAPPI estimates (figures 6 and 7), since drought projections are known to be strongly model-dependent and to display large decadal variability [35].

Overall, these analyses show that significant differences in regional extremes are found between a global warming of 1.5°C versus 2°C, especially for temperature extremes, but also for water cycle extremes in several regions.

## 5. Biogeophysical impacts of land–climate interactions and land use in low-emissions scenarios

IAM scenarios integrate land-use changes in relation to changes in crop production, CO_2_ exchanges and energy production. However, IAMs generally consider neither the impacts of warmer climates on crop production, nor the biogeophysical impacts of these land-use modifications, which are related to changes in albedo, water exchanges and momentum fluxes [36,37]. (Isolated studies consider some of these effects (e.g. [38]), but these are not integrated in scenarios feeding into the upcoming sixth assessment report of the IPCC.)

Despite their lack of consideration in the development of standard socio-economic pathways, biogeophysical effects of land use and land processes on climate are substantial, in particular at regional scale. We highlight hereafter recent evidence showing the role of regional land–climate feedbacks and land forcing in low-emissions scenarios, which could mean that two scenarios identified as leading to the same greenhouse gas-related forcing in 2100 could still result in very different regional climate impacts.

Soil moisture–climate interactions are an important driver of regional changes in climate [12,13]. This is related to the impact of soil moisture on turbulent flux partitioning in transitional climate regions [11,12,39]. In these regions, which are located at the transition between dry and wet climates (e.g. mid-latitude regions, but also some regions in the tropics [12,40]), soil moisture variability directly affects evapotranspiration, and thus also the latent and sensible heat fluxes. As a consequence, time periods with high soil moisture limitation, e.g. related to seasonal or occasional drought occurrence, display a high increase in air temperature. This mechanism has been shown to play an important role for the occurrence of hot extremes in both present and future climate [11,12], and was also identified in observational analyses [40–42].

**Figure 8.**
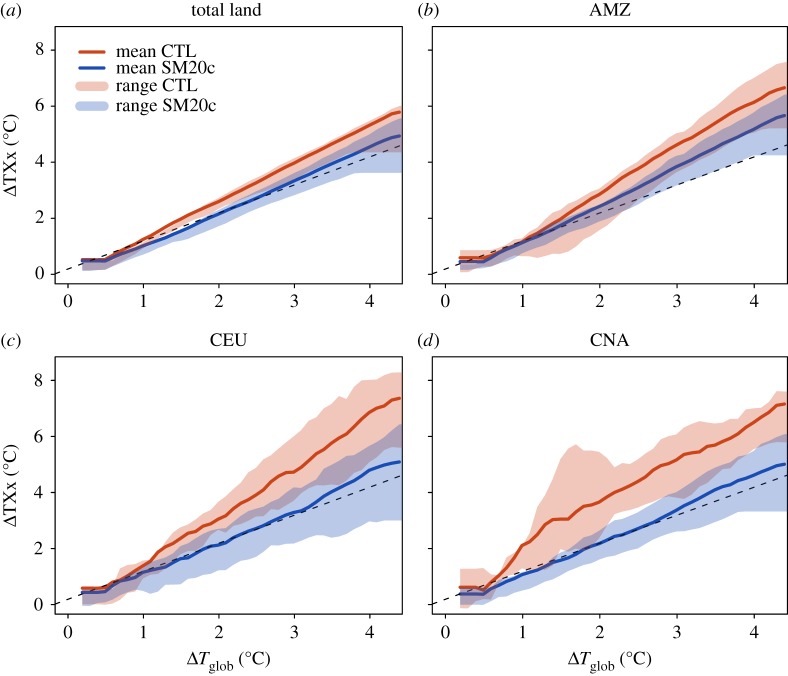
Land TXx/regional TXx anomalies versus global mean temperature anomalies based on analyses from the GLACE-CMIP5 [13] experiment for the global land and three IPCC SREX [33] regions (see text for more details and figure 2 for definition of regions). The solid lines are the multi-model means of control (CTL, red) and prescribed twentieth-century soil moisture (SM20c, blue) simulations with shadings representing their ranges of minimum and maximum values. The identity line indicates identical TXx anomaly and *T*_glob_ anomaly increase (black dashed). Anomalies are calculated as 20-year running means from 1971 to 2100 relative to the base period of 1951–1970, and added to an offset for the mean global warming in 1951–1970 compared to the pre-industrial (1861–1880) reference (same offset on *x* and *y* axes). (Adapted from [14].)

Recently, Vogel *et al*. [14] have analysed the role of soil moisture–temperature feedbacks for the relationship of temperature extremes with changes in global mean temperature derived using the ESR approach. Thereby, they used simulations from the CMIP5 – Global Land–Atmosphere Coupling Experiment (GLACE-CMIP5) [13,43]. Figure 8 shows adapted analyses from that study for the global land and three example SREX regions (AMZ, CEU, CNA; see figure 2). It displays ESR relationships for changes in the annual maximum daytime temperature (TXx) as a function of changes in global mean temperature derived from two sets of multi-model simulations: control (CTL) simulations, which correspond to the standard CMIP5 simulations, and modified experiments with prescribed twentieth-century soil moisture (SM20c).

These analyses reveal some intriguing findings. In many mid-latitude regions found to display a particularly large warming of hot extremes [5], the amplified warming disappears when assessing simulations without soil moisture modifications (figure 8). This suggests that much of the amplified warming of hot extremes in transitional climate regions is due to soil moisture feedbacks, and that, if these effects are removed, warming in these regions would be closer to the global mean temperature warming.

This has important implications for climate projections and IAM scenarios. First, it implies that processes that affect the global transient climate response (TCRglob), i.e. the change in global mean temperature, are different from those affecting the regional response of extremes (TCRreg). Second, for low-emissions scenarios, the effects of soil moisture feedbacks can have larger implications for regional changes in temperature extremes than a change in the global mean temperature of *ca* 0.5°C. This means that the minimization of regional impacts requires the consideration of regional feedbacks from land processes, in particular related to water availability. This is particularly critical, since the regions displaying strong soil moisture–temperature feedbacks include many densely populated regions in North America, Europe and Asia [5,12,13].

**Figure 9.**
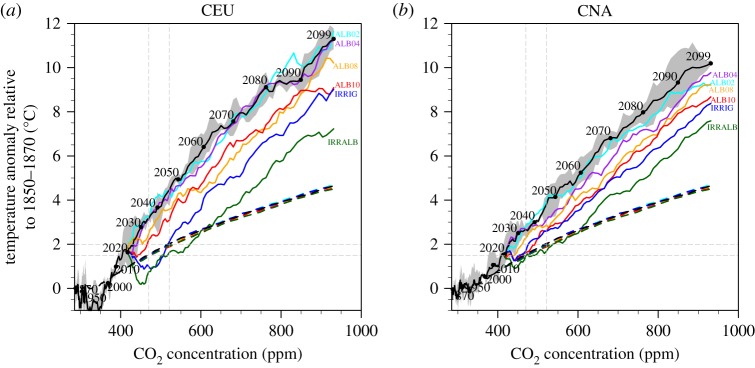
Regional temperature scaling with CO_2_ concentration (ppm) over 1850 to 2099 in NCAR CESM simulations including various land-use-based modifications for two different SREX regions: (*a*) CEU, (*b*) CNA. Solid lines correspond to the regional average annual maximum daytime temperature (TXx) anomaly, and dashed heavy lines correspond to the global mean temperature anomaly, where all temperature anomalies are relative to 1850–1870 and units are in °C. The black line in both panels denotes the three-member control ensemble mean with the grey-shaded regions corresponding to the ensemble range. The coloured lines correspond to the three-member ensemble means of experiments modifying crop albedo by 0.02 (ALB02, cyan), 0.04 (ALB04, purple), 0.08 (ALB08, orange) or 0.1 (ALB10, red), integrating irrigation in crop areas (IRRIG, blue), and combining both irrigation and changes in albedo by 0.1 (IRRALB, green). The light dashed grey lines indicate conditions at global warming levels of 1.5°C and 2°C, respectively. (Adapted from [15].)

As a further illustration of the key role of biogeophysical land–atmosphere interactions for low-emissions scenarios, we also consider recent results from Hirsch *et al*. [15] (figure 9), which assessed the impact of conceptual land-use scenarios in global climate model projections (computed with the Community Earth System Model (CESM) version 1.2 [44]). The experiments include seven configurations consisting of a control (CTL) with no land-use modifications, four experiments with different levels of crop albedo enhancement, +0.02, +0.04, +0.08 and +0.10 (denoted as ALB02, ALB04, ALB08 and ALB10, respectively), one experiment with irrigation enabled (IRRIG), and finally one experiment with both crop albedo enhancement of +0.10 and irrigation enabled (IRRALB) to evaluate the level of complementarity between the two modifications. The crop albedo modifications are only applied when the leaf area index is non-zero, and only to the canopy albedo and not the soil albedo. Such increases in cropland albedo could be induced by the (possibly combined) application of conservation agriculture (no-till farming), the implementation of more light-reflective crops and other land-use management practices, whereby an increase of 0.1 represents an upper bound [15,45–47]. The irrigation is applied to the entire crop fraction but only when crops are the dominant vegetation cover within the given grid cell and when the conditions for triggering irrigation (i.e. growing season and water stress) are met. The experiments remain highly idealized because the respective modifications are applied globally to the whole crop area. Nonetheless, they assess a potential upper bound for the impact resulting from land-use modifications in a state-of-the-art ESM.

The results displayed in figure 9 reveal again a very high impact of the land-based conditions on temperature extremes (TXx) in two regions with particularly strong warming of hot extremes (figure 5), Central Europe (CEU) and Central North America (CNA). Consistent with the results from Vogel *et al*. [14] which suggest that much of the amplified warming of hot extremes is due to soil moisture feedbacks (figure 6), the CESM simulations including irrigation (and thus reduced soil moisture limitation) over all of the cropland area display a much reduced warming (blue line). We note that changes in albedo are also found to have some non-negligible effect on hot extremes, in particular for albedo increases of 0.1, consistent with other analyses [45,47]. Nonetheless, their effect appears smaller than that of irrigation in the considered (idealized) experiment.

An important feature also highlighted in Hirsch *et al*. [15] is the fact that the total effect of irrigation or albedo on the regional temperatures is found to be almost independent of the level of global warming (close to constant offset from the line for the CTL experiment; figure 9). This means that, in relative terms, the changes in land-based conditions play a much more important role for low-emissions rather than high-emissions scenarios. For this reason, considering such feedbacks in the development of ambitious mitigation scenarios is essential.

We note that current ESMs generally do not integrate irrigation in projections, while the land-use changes simulated by IAMs imply large increases in cropland area (see §6), and therefore also of irrigation. Hence, especially the role of irrigation and its possible feedbacks to regional climate is likely to be particularly important. In addition, the required water amounts implied by some of the cropland expansions could exceed sustainability limits and would need to be carefully evaluated [48].

**Figure 10.**
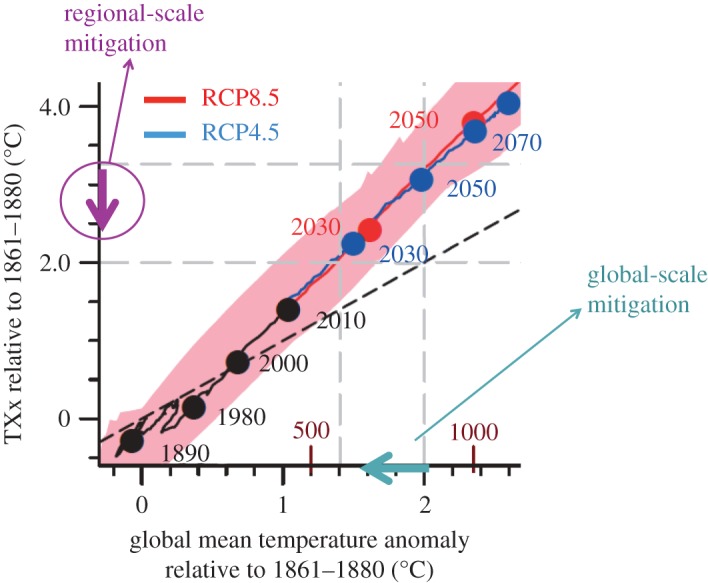
Role of global-scale versus regional-scale mitigation. Schematics based on the ESR-CMIP5 relationship of TXx versus change in global mean temperature in the Mediterranean region (from [5], see also figure 1), with zoom on changes up to 2°C of global warming. Reduced regional impacts can be achieved both through a decrease in the global mean temperature (light blue arrow, ‘global-scale mitigation’), as well as through a reduction in the regional sensitivity of temperature extremes to global temperature changes (violet arrow, ‘regional-scale mitigation’).

The important role of regional processes in mitigation is further illustrated in the schematic displayed in figure 10, showing, for example, that changes in hot temperature extremes in the Mediterranean region can be decreased not only through a mitigation of the global temperature warming (light blue arrow), but also through a decrease of the regional sensitivity of extremes to global warming (‘regional-scale mitigation’, violet arrow).

## 6. Land-use changes in IAM simulations

Hereafter, we briefly assess the range of changes in land use simulated in current state-of-the-art IAMs (see §2c for details and reference publications for the considered models). The considered IAMs include GCAM, MESSAGE-GLOBIOM, IMAGE and REMIND-MAgPIE.

**Figure 11.**
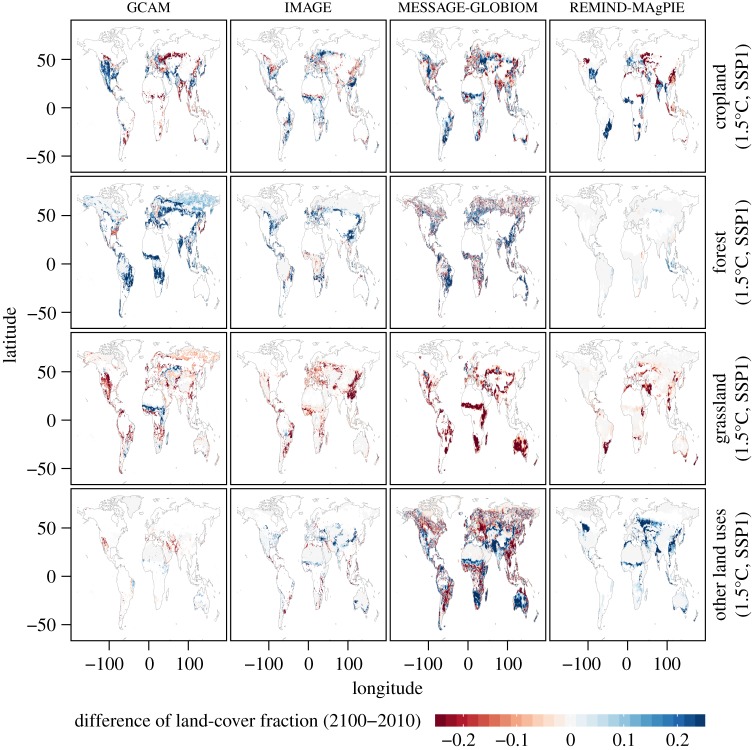
Difference of land-cover fractions between 2100 and 2010 from the GCAM, IMAGE, MESSAGE-GLOBIOM and REMIND-MAgPIE IAMs grouped by land-cover type for 1.5°C of global warming and SSP1 scenario. Grid cells are masked if the land-cover fraction is less than or equal to 1%. The values are displayed as differences in fractional areas (unitless).

**Figure 12.**
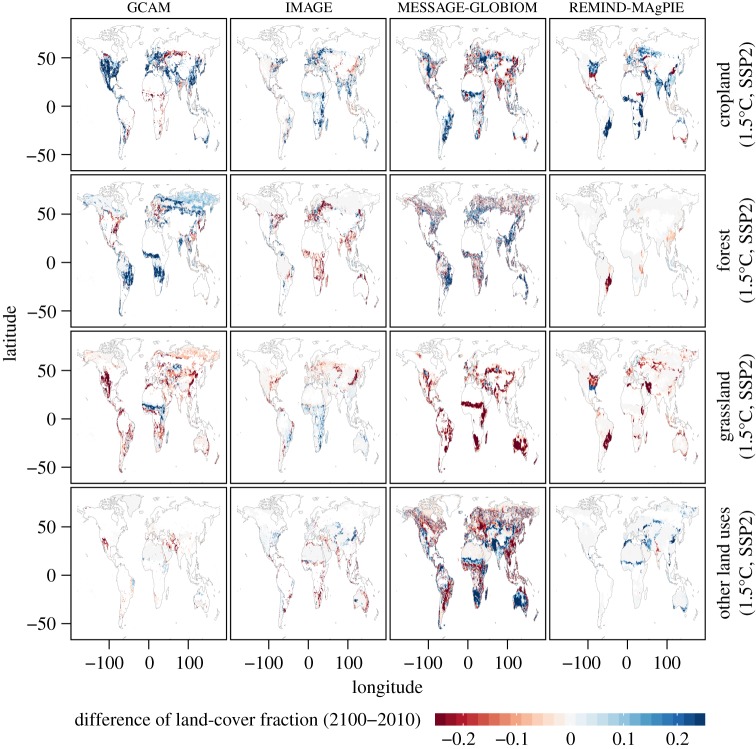
Difference of land-cover fractions between 2100 and 2010 from the GCAM, IMAGE, MESSAGE-GLOBIOM and REMIND-MAgPIE IAMs grouped by land-cover type for 1.5°C of global warming and SSP2 scenario. Grid cells are masked if the land-cover fraction is less than or equal to 1%. The values are displayed as differences in fractional areas (unitless).

Figures 11 and 12 display the differences in land-cover fractions between 2100 and 2010 from the four considered IAMs (see §2d) for 1.5°C of global warming and the SSP1 and SSP2 scenarios. Scenarios for 2°C of global warming show similar differences in patterns, though generally with less substantial land-use changes (LUCs; not shown). Summary statistics are also given in table 4.

**Table 4.** Percentages of land-cover coverage in IAM scenarios, for 2010 and 2100 (at 1.5°C warming) and SSP1 and SSP2 scenarios ([sum of land-cover fraction]/[sum of all land grid cells]).

mapped land-cover class	GCAM (PNNL)	MESSAGE-GLOBIOM (IIASA)	IMAGE (PBL)	REMIND-MAgPIE (PIK)
cropland	2010, SSP1: 9.8	2010, SSP1: 10.7	2010, SSP1: 10.4	2010, SSP1: 9.8
	2010, SSP2: 9.8	2010, SSP2: 10.7	2010, SSP2: 10.4	2010, SSP2: 9.9
	2100, SSP1: 9.6	2100, SSP1: 13.0	2100, SSP1: 11.7	2100, SSP1: 8.9
	2100, SSP2: 14.5	2100, SSP2: 13.6	2100, SSP2: 13.5	2100, SSP2: 13.4
grassland	2010, SSP1: 34.3	2010, SSP1: 10.6	2010, SSP1: 19.7	2010, SSP1: 19.9
	2010, SSP2: 34.3	2010, SSP2: 10.6	2010, SSP2: 19.7	2010, SSP2: 19.9
	2100, SSP1: 28.5	2100, SSP1: 4.1	2100, SSP1: 14.7	2100, SSP1: 15.9
	2100, SSP2: 26.8	2100, SSP2: 4.4	2100, SSP2: 19.2	2100, SSP2: 15.1
forest	2010, SSP1: 32.3	2010, SSP1: 22.6	2010, SSP1: 35.4	2010, SSP1: 31.0
	2010, SSP2: 32.3	2010, SSP2: 22.6	2010, SSP2: 35.3	2010, SSP2: 31.0
	2100, SSP1: 38.8	2100, SSP1: 26.2	2100, SSP1: 39.7	2100, SSP1: 31.2
	2100, SSP2: 35.7	2100, SSP2: 26.0	2100, SSP2: 34.5	2100, SSP2: 30.5
other land and urban	2010, SSP1: 17.9	2010, SSP1: 51.5	2010, SSP1: 29.0	2010, SSP1: 32.3
	2010, SSP2: 17.9	2010, SSP2: 51.5	2010, SSP2: 29.0	2010, SSP2: 32.2
	2100, SSP1: 17.2	2100, SSP1: 52.1	2100, SSP1: 28.3	2100, SSP1: 36.9
	2100, SSP2: 16.8	2100, SSP2: 51.3	2100, SSP2: 27.3	2100, SSP2: 34.0

Dominant features of LUCs in the four IAMs can be identified, as well as substantial variations between IAMs. Overall, all IAMs display substantial decreases in grassland area, which is replaced by cropland and/or forest cover (and in some cases also other land uses). The IAMs differ substantially in how they allocate the forest and cropland areas, with some cropland areas being replaced by forest or vice versa. These differences also depend strongly on the considered SSP scenario, with SSP1 favouring increases in forest cover, and SSP2 increases in cropland.

It is important to consider the LUC patterns of the IAMs individually, since there are substantial differences between IAMs (see also table 4). While all IAMs display some decreases in grassland area, these are much less pronounced for the IMAGE SSP2 scenario, and in part also the REMIND-MAgPIE scenarios. The changes in forest area are very different in the four considered IAMs, with some widespread increases in GCAM and MESSAGE-GLOBIOM, but no changes in REMIND-MAgPIE, and both smaller increases (SSP1) and some decreases (SSP2) in IMAGE. The regional patterns of changes in cropland are also extremely different in the IAMs. For instance, GCAM simulates large increases in cropland extent in North America, including in the dry western part of the USA, while the other three models do not display any increase in cropland in the latter region.

To further interpret this spread in the IAM land-use maps, we also consider the actual 2100 maps of land-cover fractions for the single IAMs in the electronic supplementary material (figures S15–S18). The maximum range in land fraction and the respective IAM models that present the highest or lowest fraction for a given land class in each location for the considered SSP and global warming scenarios by 2100 are also displayed (electronic supplementary material, figures S19–S21). Some further systematic differences are found. For instance, the IMAGE model tends to include more forest cover in high latitudes, independently of the considered scenario. GLOBIOM on the other hand includes very little grassland.

Overall, the analyses show that each model has distinctive characteristics with respect to land-use allocation and that the inter-IAM spread is substantial, both with respect to static maps, as well as with respect to changes over the course of the twenty-first century. Some of these differences reflect the fact that global land allocation for and the distribution of dedicated bioenergy plantations (with or without combination with carbon capture and storage (CCS)) in IAMs for a fixed level of carbon sequestration by 2050 or 2100 highly depends on assumptions being made on socio-economic, climate and environmental policy as well as technological developments [49]. Thereby, less or more land might be needed in IAMs to fulfil a certain climate target. In addition, differences in observational data used as input to the models also probably play a role [50].

These differences in IAMs for resulting LUCs corresponding to the same SSP and climate scenarios reveal an additional dimension of uncertainty that has not been considered in multi-model projections so far. Indeed, biogeophysical processes associated with the respective land uses may lead to very different regional climate conditions if forest cover is, for instance, included instead of crops or grassland (e.g. [16,51–53]), if cropland is expanded in regions requiring irrigation [48,54], or if albedo changes are associated with given changes in land use [47]. In addition, a recent ESM multi-model study using the IMAGE low-emissions scenarios suggests that, even for the same IAM, the consideration of the land-use spread associated with different SSP scenarios may have a higher impact on climate than a difference of 0.5°C of global warming for some models and regions [16]. This finding would need to be evaluated with scenarios from other IAMs, given the large differences identified in figures 11 and 12.

Current ESMs display a substantial spread in responses to biogeophysical impacts from land use [16,37], as well as some systematic biases [55]. Hence the magnitude and direction of LUCs under low-emissions scenarios, as well as the resulting responses in ESMs, remain a large source of uncertainty, which could substantially affect the identification of desirable mitigation and adaptation pathways. Some aspects of this question will also be addressed in an upcoming CMIP6 experiment, the ‘Land Use Model Intercomparison Project’ (LUMIP) [56].

## 7. Conclusion

In this article, we have assessed changes in temperature and water cycle extremes at 1.5°C versus 2°C of global warming, including a perspective on the impacts of land processes and land-use changes on these projections.

New analyses comparing ESR-derived estimates of changes in extremes at 1.5°C and 2°C of global warming with output from simulations of the HAPPI multi-model experiment reveal that the two approaches yield similar overall results. This suggests that the transient response of regional climate extremes to global warming anomalies is fairly consistent with that of responses at semi-equilibrium, when the sea surface temperatures (and sea ice extent) are fixed to conditions associated with these respective warming levels in projections for the end of the twenty-first century.

We further highlight that the regional amplification of hot extremes identified in many land regions with transitional conditions between dry and wet climates [5] can be related to soil moisture feedbacks [14]. As a consequence, any regional biogeophysical modifications of land processes, e.g. through land-use changes affecting land-cover type or land management—and thereby albedo or moisture fluxes—are found to strongly affect these regional changes in climate extremes, especially for low-emissions scenarios [15,16,47]. This result is critical for the development of future climate projections and IAM scenarios, since biogeophysical feedbacks of simulated changes in land use are not considered in the development of IAM models and could modify the identified optimal mitigation pathways, in particular for low-emissions scenarios.

The identified strong effect of land-based forcing on regional extremes may also question the finding that the ESR estimates of the sensitivity of changes in regional extremes to changes in global warming appears mostly independent of the considered emissions scenario in CMIP5 experiments [5,8]. Indeed, the lack of sensitivity of the regional ESR to the considered emissions scenarios for projections of temperature and precipitation extremes may reflect an intrinsic property of the climate system [20,57], but it may also reflect a lack of diversity in the regional details of the considered emissions scenarios within CMIP5. It is likely that more diverse projections which would consider the full (global and regional) spread of possible land-use scenarios resulting from IAMs and include biogeophysical effects of these land-use conditions would lead to a larger spread of ESR responses between the considered scenarios. A particularly important aspect is the potential role of irrigation, which is not integrated in present-day ESMs but could strongly affect temperature means and extremes in both present and future climate [15,54].

In conclusion, we find that the somewhat *ad hoc* ESR procedures developed to assess changes in climate at 1.5°C versus 2°C and higher levels of warming are fairly robust when compared with actual simulations prescribing ocean temperature forcing consistent with these warming levels. This is a helpful result in view of the upcoming IPCC SR15 report, which was developed under a high time constraint and for which dedicated model experiments were mostly non-existent at the time of writing. However, we also highlight that the role of biogeophysical land-based forcing for regional changes in climate extremes and impacts is not accounted for in the development of reference IAM-based scenarios to date, nor in the majority of modelling experiments assessing changes in climate at 1.5°C versus higher levels of warming. Future investigations will be needed to better quantify the impact of changes in land use for regional projections in low-emissions scenarios and the extent to which they affect derived sustainable development pathways.

## Supplementary Material

Supplementary Information

## Data accessibility

The data underlying figures 3–7 can be obtained from S.I.S. (sonia.seneviratne@ethz.ch) and R.W. (richard.wartenburger@env.ethz.ch). The IAM land-use maps underlying figures 11 and 12 can be obtained from the GCAM (katherine.calvin@pnnl.gov), IMAGE (IMAGE-info@pbl.nl), MESSAGE-GLOBIOM (havlikpt@iiasa.ac.at, krisztin@iiasa.ac.at) and REMIND-MAgPIE (popp@pik-potsdam.de) modelling teams. Electronic supplementary material is available online at rs.figshare.com.
