# Supplementary material for: Climate extremes, land–climate feedbacks and land-use forcing at 1.5°C
Source: Philos Trans A Math Phys Eng Sci. 2018 Apr 2;376(2119):20160450. doi: 10.1098/rsta.2016.0450 (PMC5897823; doi:10.1098/rsta.2016.0450)
Supplement: Supplementary Information [file rsta20160450supp1.pdf]

Climate extremes, land-climate feedbacks, and land use forcing at 1.5°C - Supplementary Information

Sonia I. Seneviratne[1], Richard Wartenburger[1], Benoit P. Guillod[1,2], Annette L. Hirsch[1], Martha M. Vogel[1], Victor Brovkin[3], Detlef P. van Vuuren[4,5], Nathalie Schaller[6], Lena Boysen[3], Katherine V. Calvin[7], Jonathan Doelman[4], Peter Greve[8], Petr Havlik[8], Florian Humpenöder[9], Tamas Krisztin[8], Daniel Mitchell[10], Alexander Popp[9], Keywan Riahi[8], Joeri Rogelj[1,8], Carl-Friedrich Schleussner[9,11], Jana Sillmann[6], and Elke Stehfest[4]

[1]Institute for Atmospheric and Climate Science, ETH Zurich, 8092 Zurich, Switzerland
[2]Institute for Environmental Decisions, ETH Zurich, 8092 Zurich, Switzerland
[3]Max-Planck Institute for Meteorology, Hamburg, Germany
[4]PBL Netherlands Environmental Assessment Agency, P.O. Box 303, Bilthoven 3720 AH, The Netherlands
[5]Copernicus Institute, Utrecht University, Heidelberglaan 2, Utrecht, The Netherlands
[6]CICERO, Oslo, Norway
[7]Pacific Northwest National Laboratory, Joint Global Change Research Institute, College Park, MD 20740, USA
[8]International Institute for Applied Systems Analysis (IIASA), Laxenburg A-2361, Austria
[9]Potsdam Institute for Climate Impact Research (PIK), Member of the Leibniz Association, P.O. Box 60 12 03, D-14412 Potsdam, Germany
[10]University of Bristol, Bristol, UK
[11]Climate Analytics, Berlin, Germany

Phil. Trans. Royal Soc. A, Special Issue on 1.5°C

January 30, 2018

.

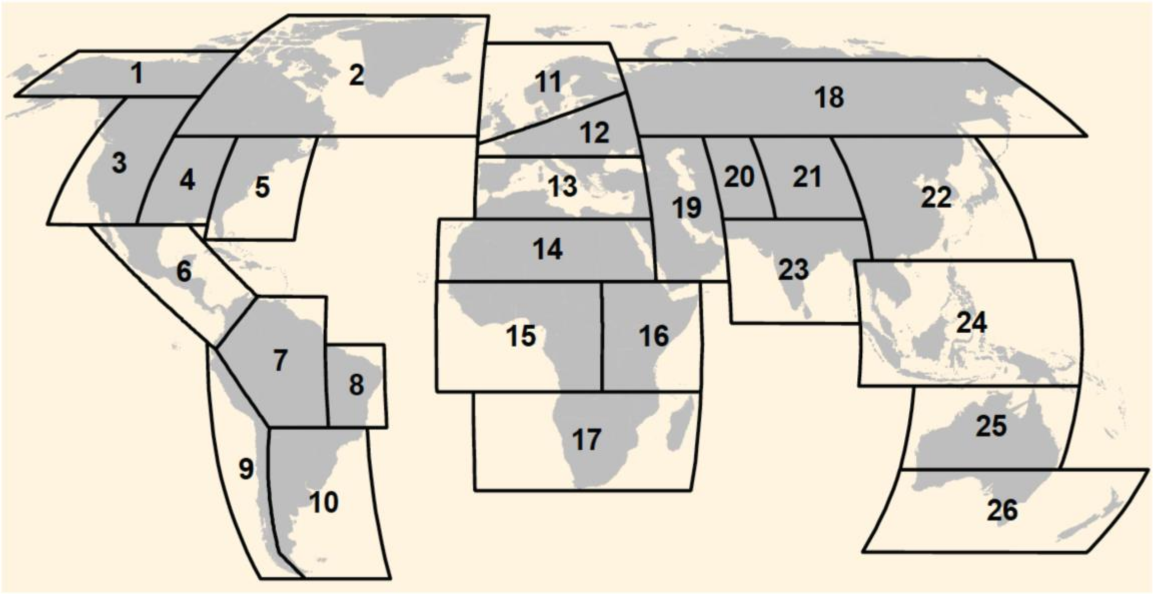

Supplementary Figure S1: IPCC SREX regions (from ref #33). 1: ALA, 2: CGI, 3: WNA, 4: CAN, 5: ENA, 6: CAM, 7: AMZ, 8: NEB, 9: WSA, 10: SSA, 11: NEU, 12: CEU, 13: MED, 14: SAH, 15: WAF, 16: EAF, 17: SAF, 18: NAS, 19: WAS, 20: CAS, 21: TIB, 22: EAS, 23: SAS, 24: SEA, 25: NAU, 26: SAU.

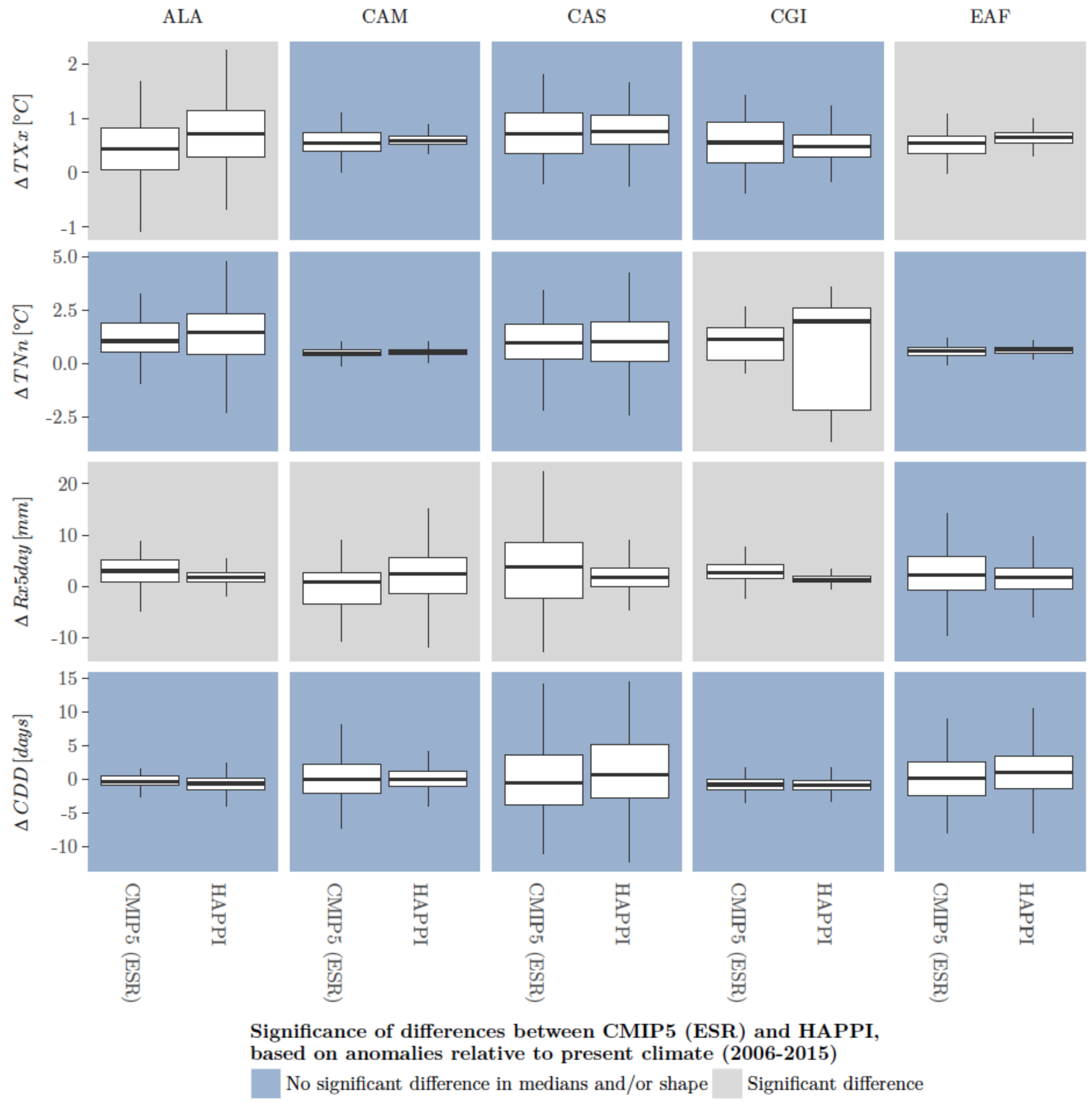

Supplementary Figure S2: Same as Fig. 3 for the SREX regions ALA (1), CAM (6), CAS (20), CGI (2) and EAF (16) (see Figure S1 for a definition of the regions). Changes in regional extremes at 1.5°C global warming estimated from the ESR approach based on CMIP5 simulations ("CMIP5 (ESR)") and from the HAPPI simulations.

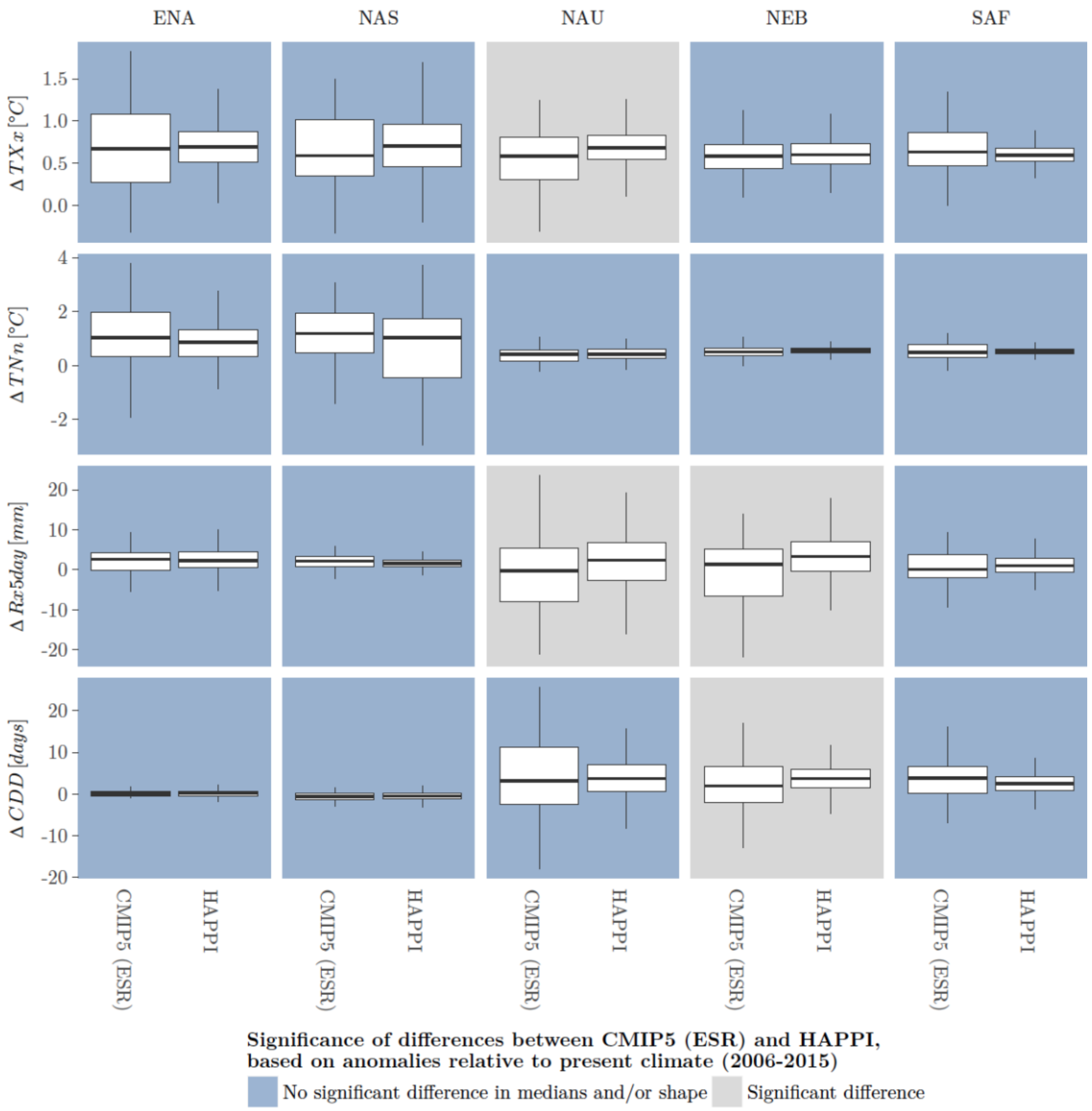

Supplementary Figure S3: Same as Fig. 3 for the SREX regions ENA (5), NAS (18), NAU (25), NEB (8) and SAF (17) (see Figure S1 for a definition of the regions). Changes in regional extremes at 1.5°C global warming estimated from the ESR approach based on CMIP5 simulations ("CMIP5 (ESR)") and from the HAPPI simulations.

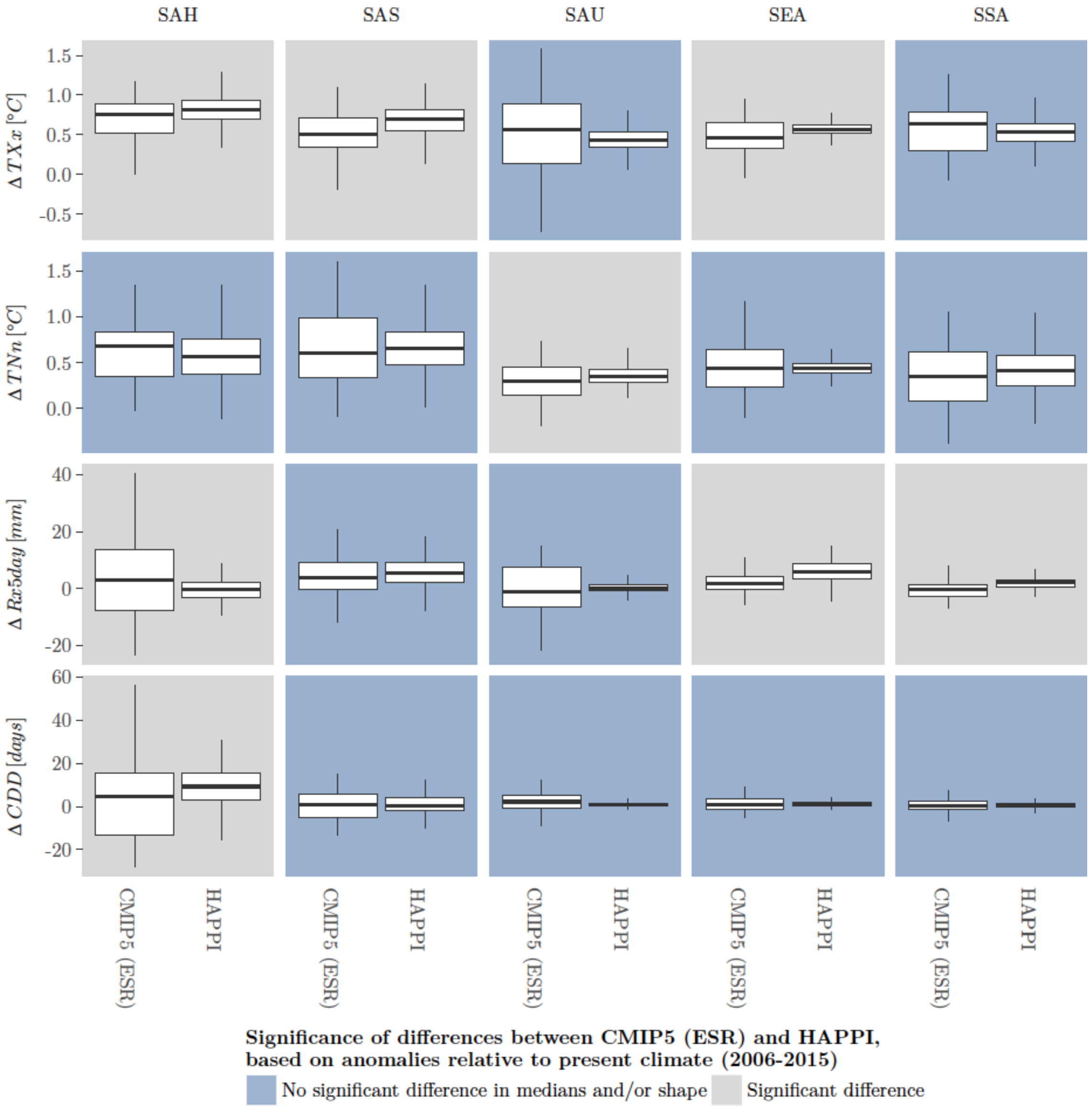

Supplementary Figure S4: Same as Fig. 3 for the SREX regions SAH (14), SAS (23), SAU (26), SEA (24) and SSA (10). (see Figure S1 for a definition of the regions). Changes in regional extremes at 1.5°C global warming estimated from the ESR approach based on CMIP5 simulations ("CMIP5 (ESR)") and from the HAPPI simulations.

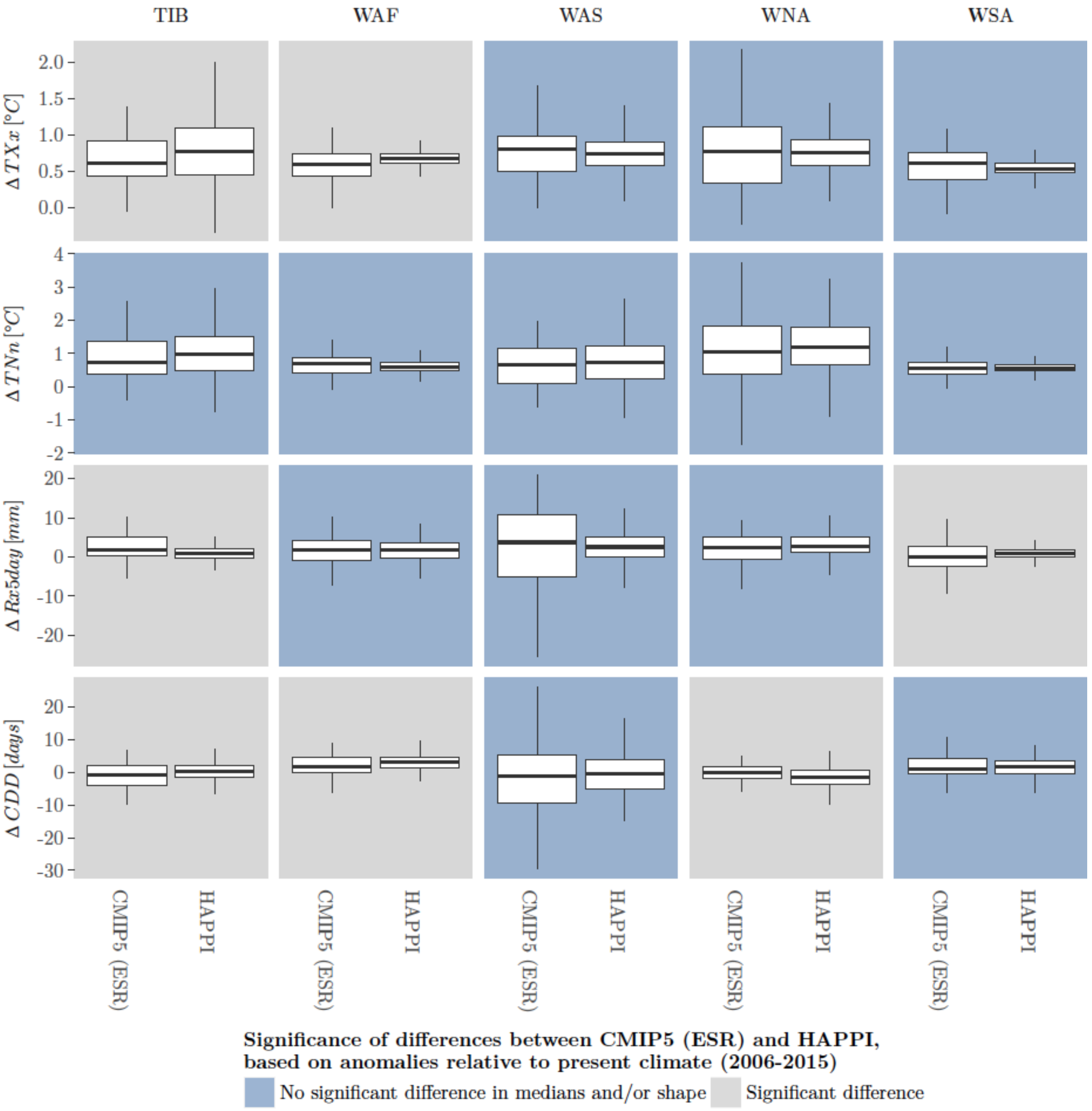

Supplementary Figure S5: Same as Fig. 3 for the SREX regions TIB (21), WAF (15), WAS (19), WNA (3) and WSA (9). (see Figure S1 for a definition of the regions). Changes in regional extremes at 1.5°C global warming estimated from the ESR approach based on CMIP5 simulations ("CMIP5 (ESR)") and from the HAPPI simulations

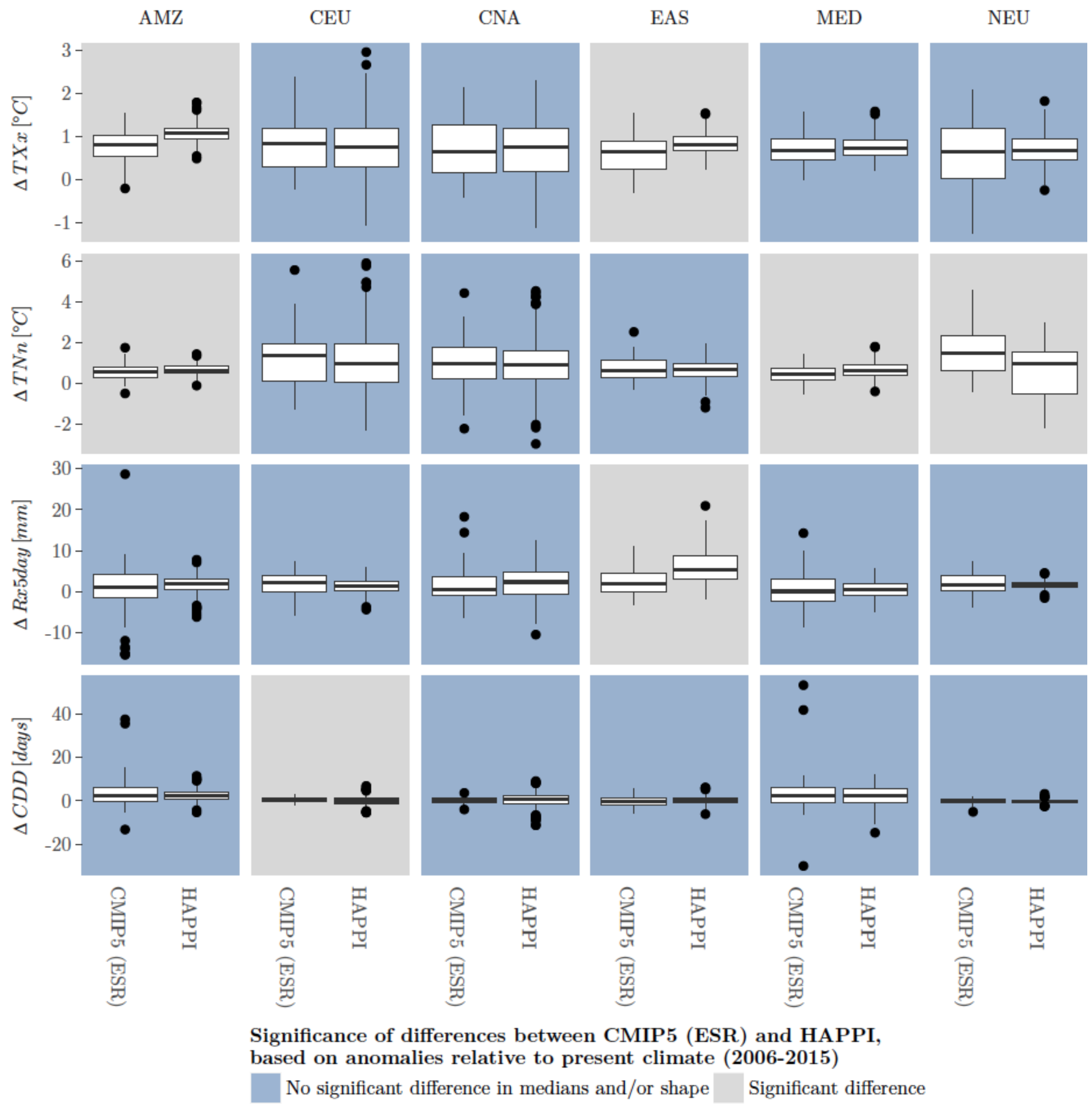

Supplementary Figure S6: Same as Fig. 3, but including outliers.

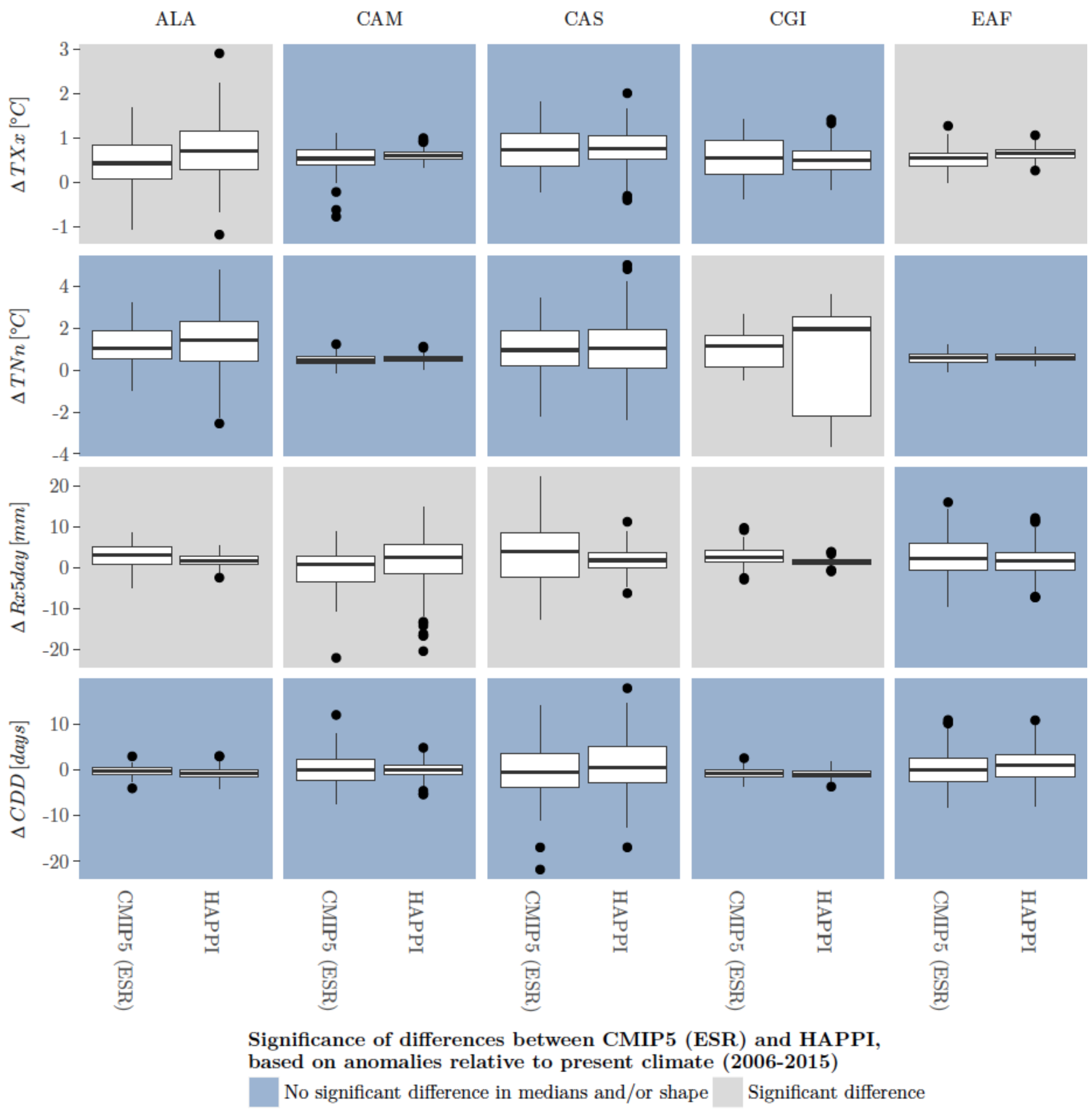

Supplementary Figure S7: Same as Fig. S2, but including outliers.

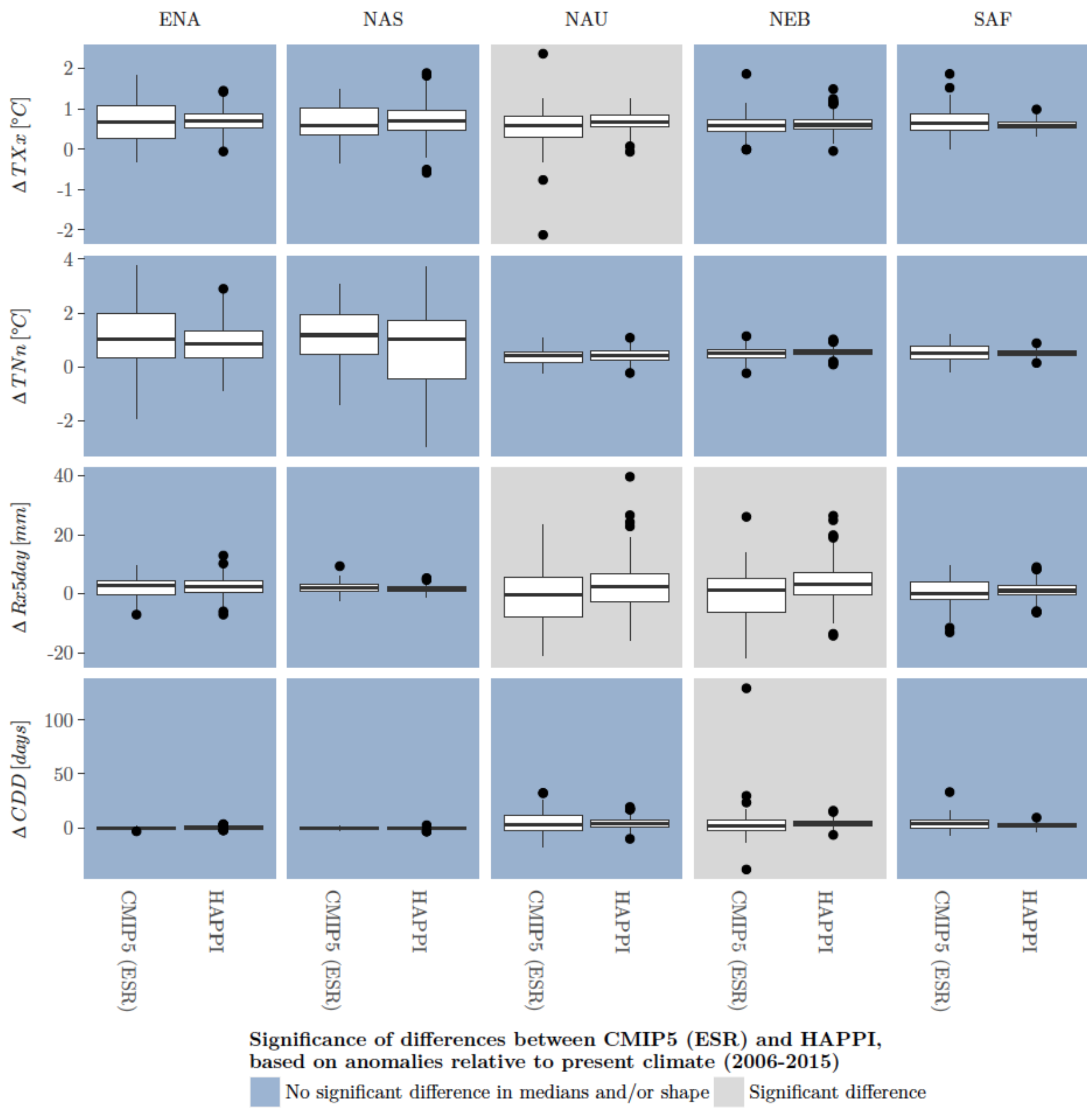

Supplementary Figure S8: Same as Fig. S3, but including outliers.

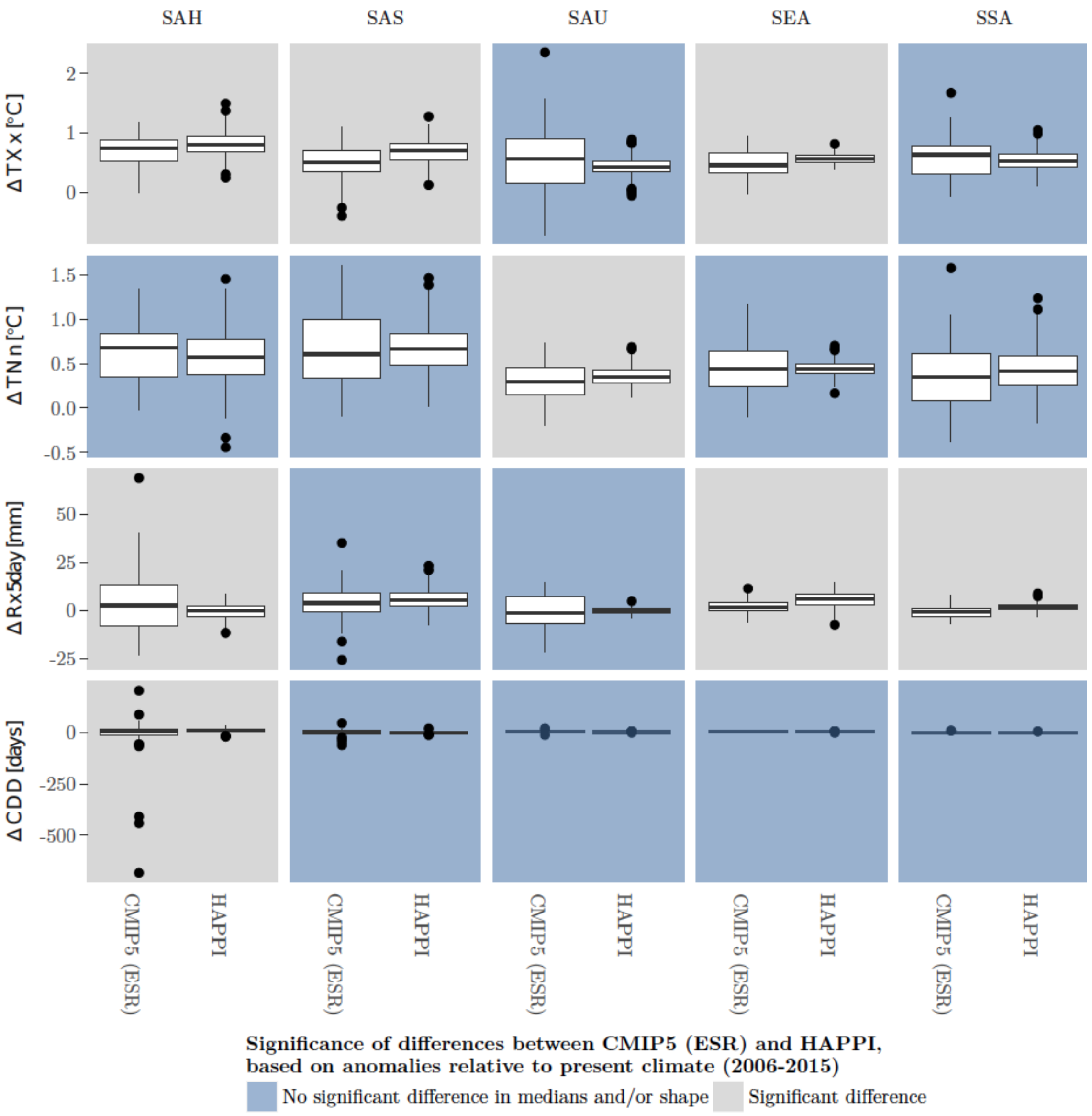

Supplementary Figure S9: Same as Fig. S4, but including outliers.

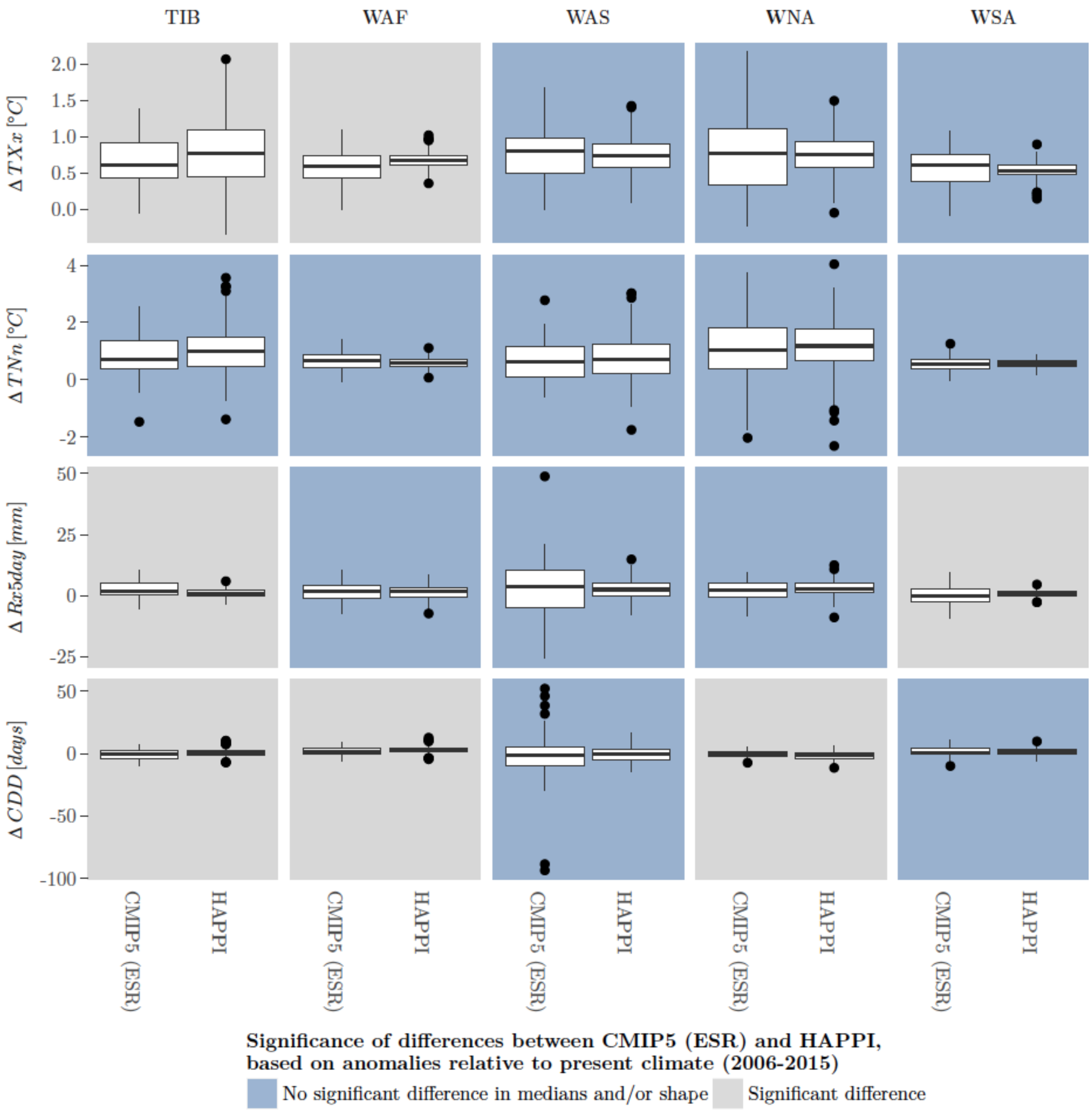

Supplementary Figure S10: Same as Fig. S5, but including outliers.

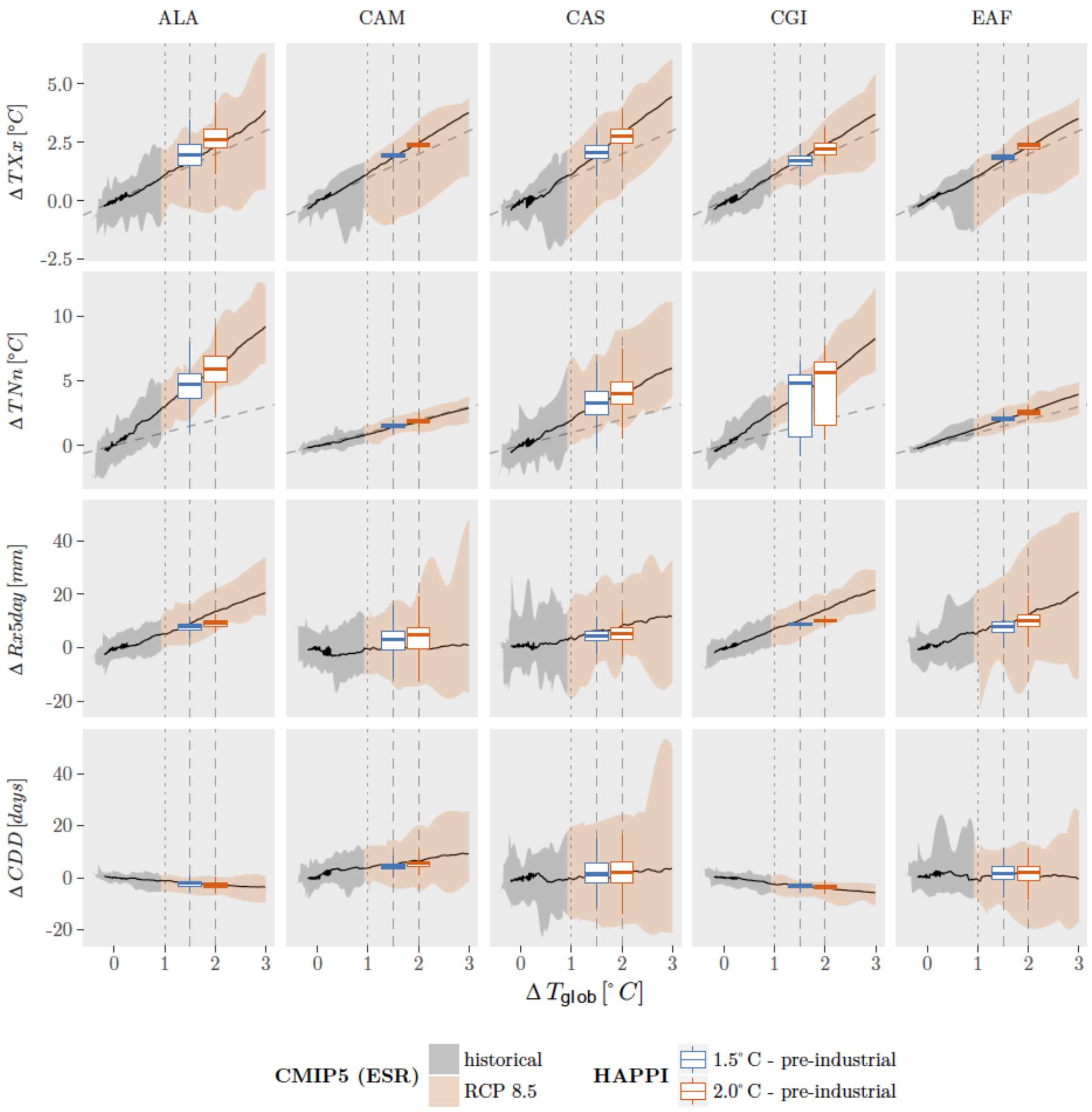

Supplementary Figure S11: Same as Fig. 5 for the SREX regions ALA (1), CAM (6), CAS (20), CGI (2) and EAF (16) (see Figure S1 for a definition of the regions).

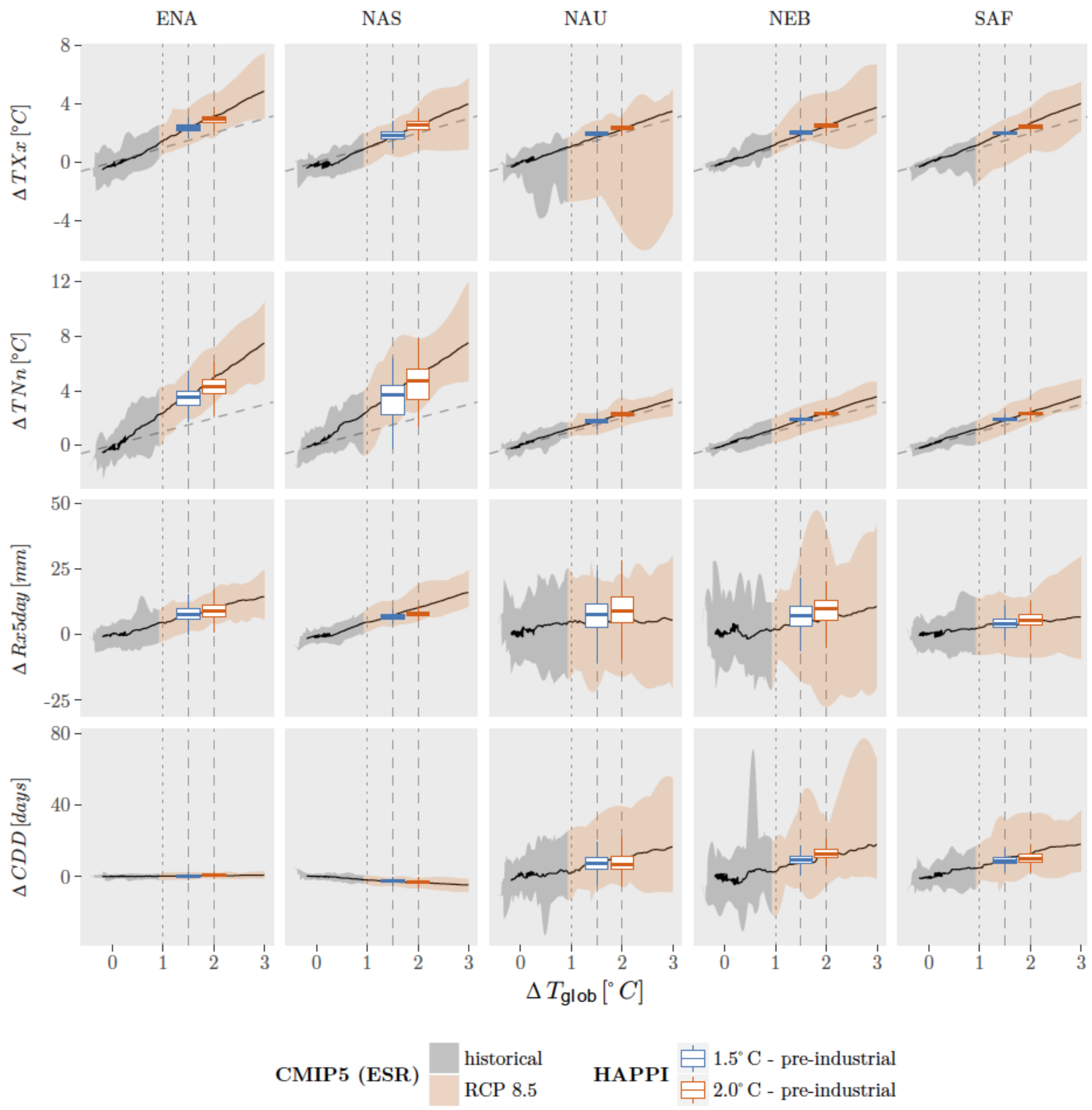

Supplementary Figure S12: Same as Fig. 5 for the SREX regions ENA (5), NAS (18), NAU (25), NEB (8) and SAF (17) (see Figure S1 for a definition of the regions).

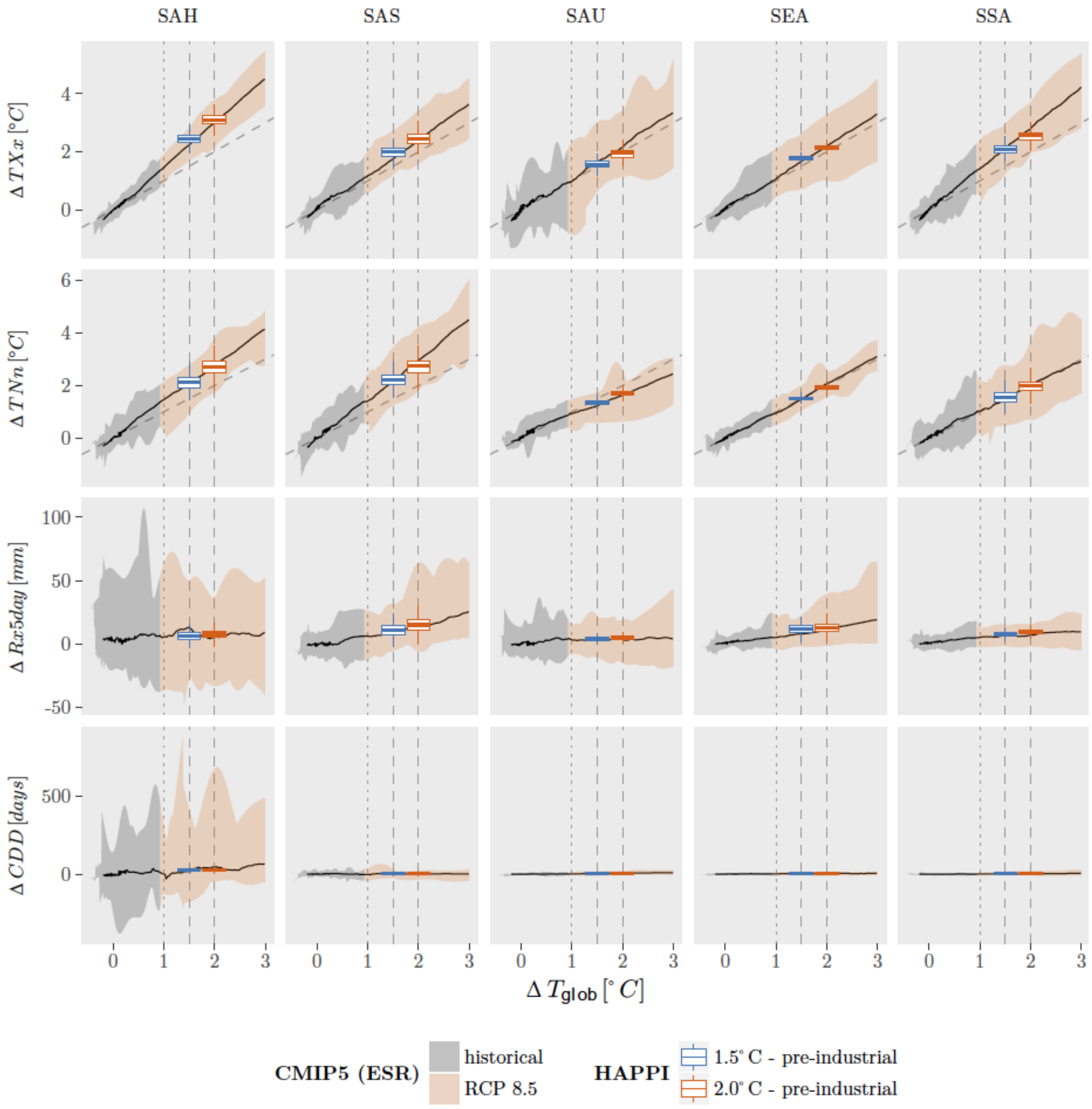

Supplementary Figure S13: Same as Fig. 5 for the SREX regions SAH (14), SAS (23), SAU (26), SEA (24) and SSA (10) (see Figure S1 for a definition of the regions).

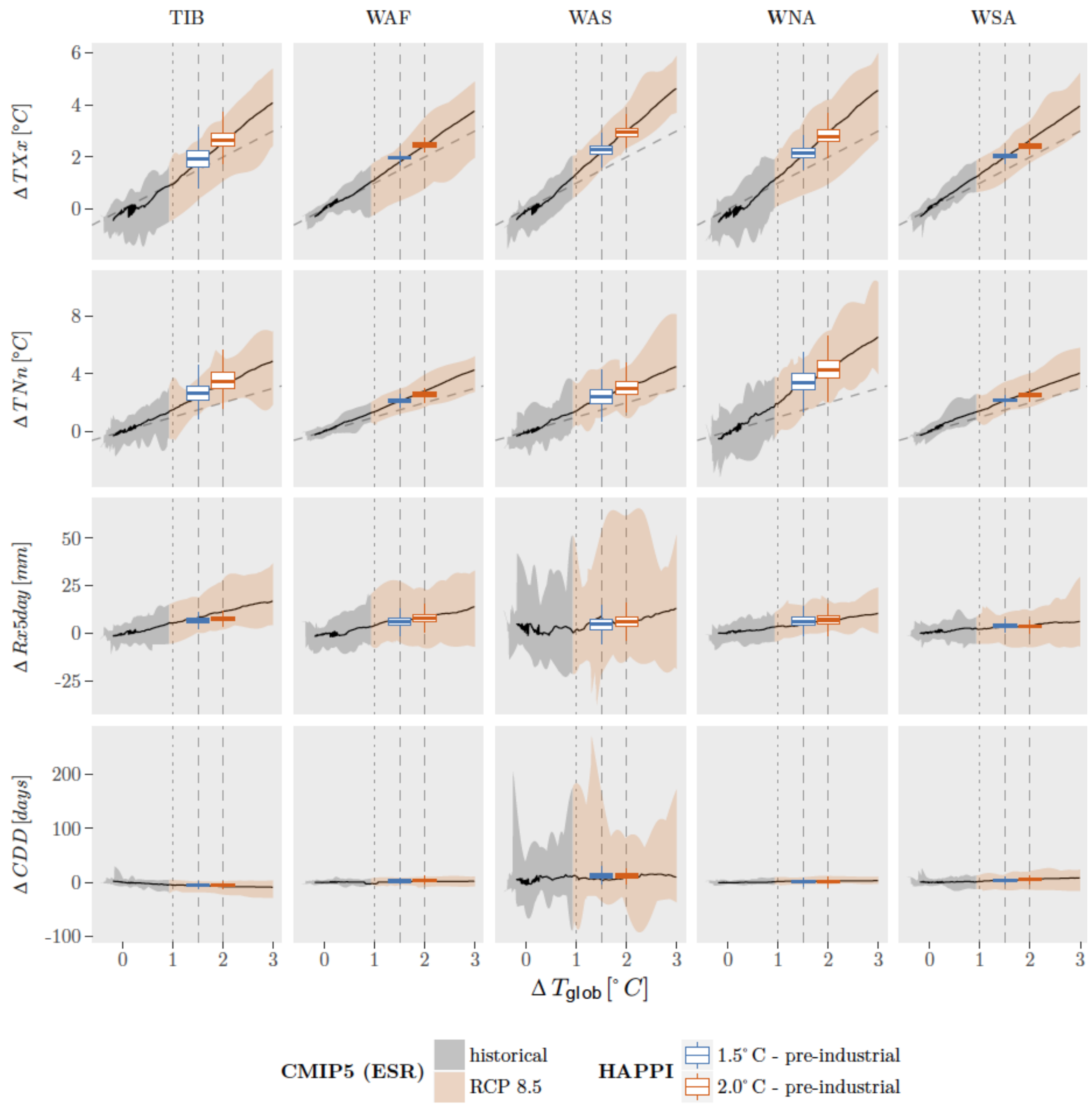

Supplementary Figure S14: Same as Fig. 5 for the SREX regions TIB (21), WAF (15), WAS (19), WNA (3) and WSA (9) (see Figure S1 for a definition of the regions).

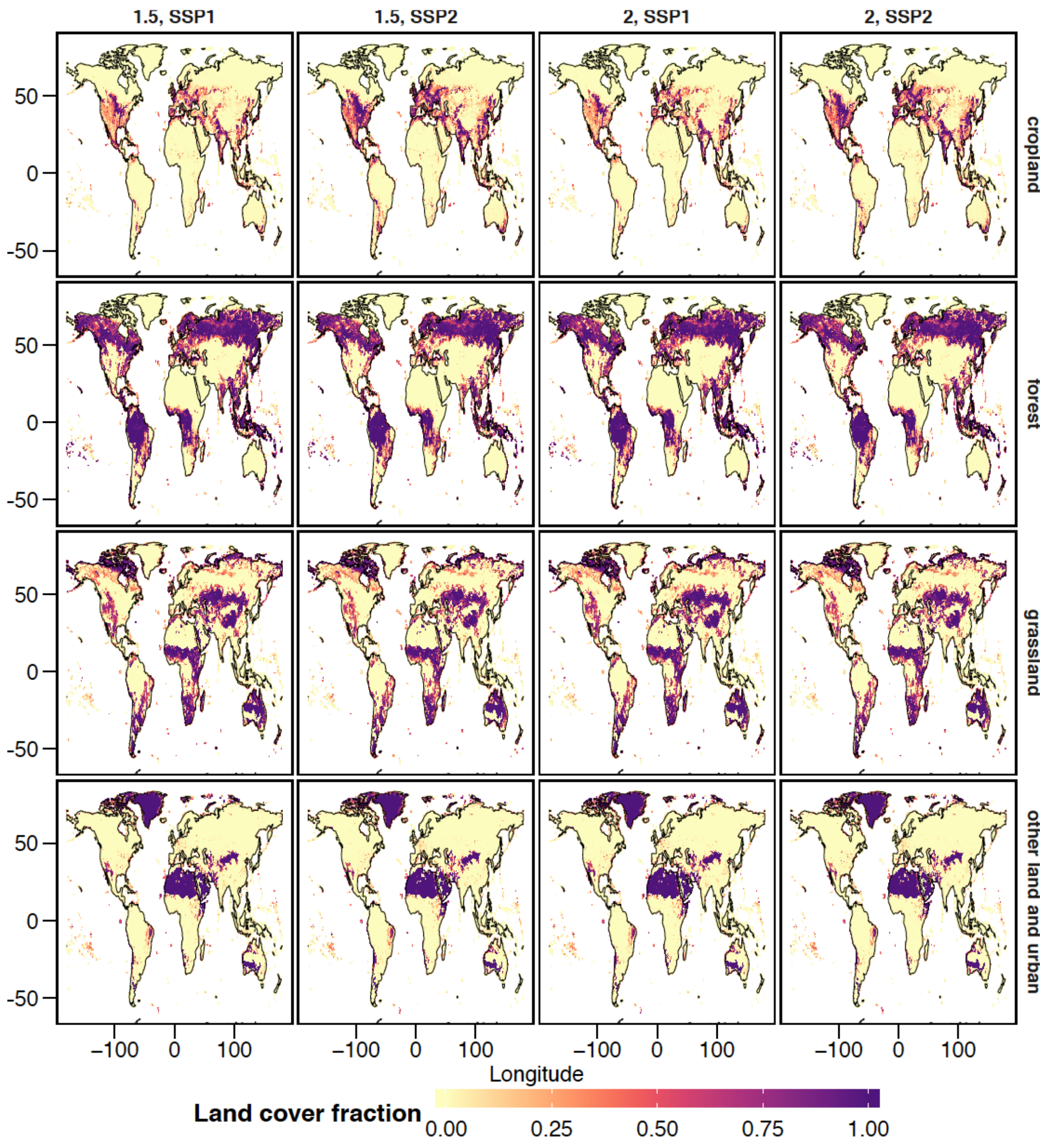

Supplementary Figure S15: Fraction of respective land cover classes in 2100 in GCAM.

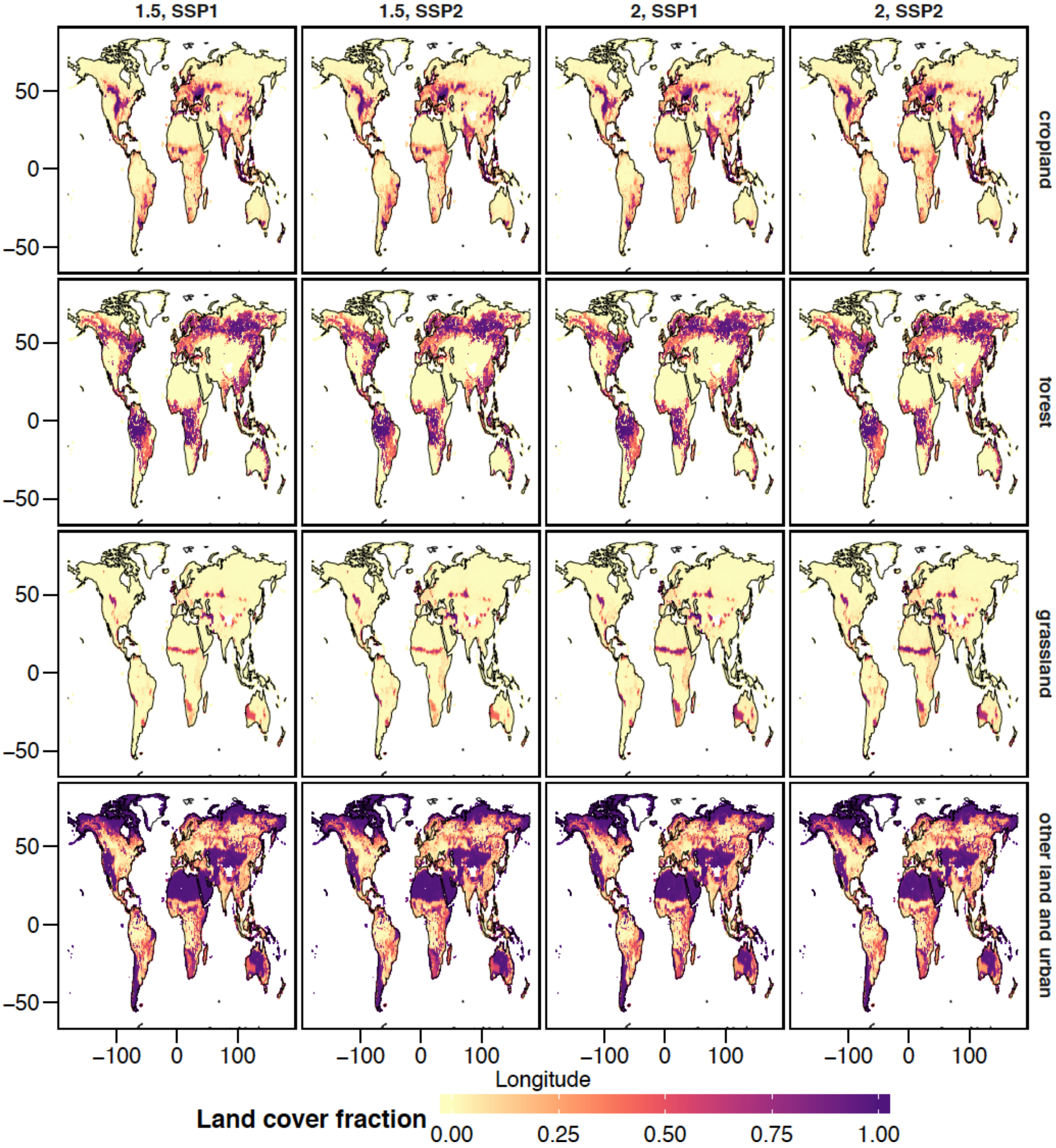

Supplementary Figure S16: Fraction of respective land cover classes in 2100 in GLOBIOM.

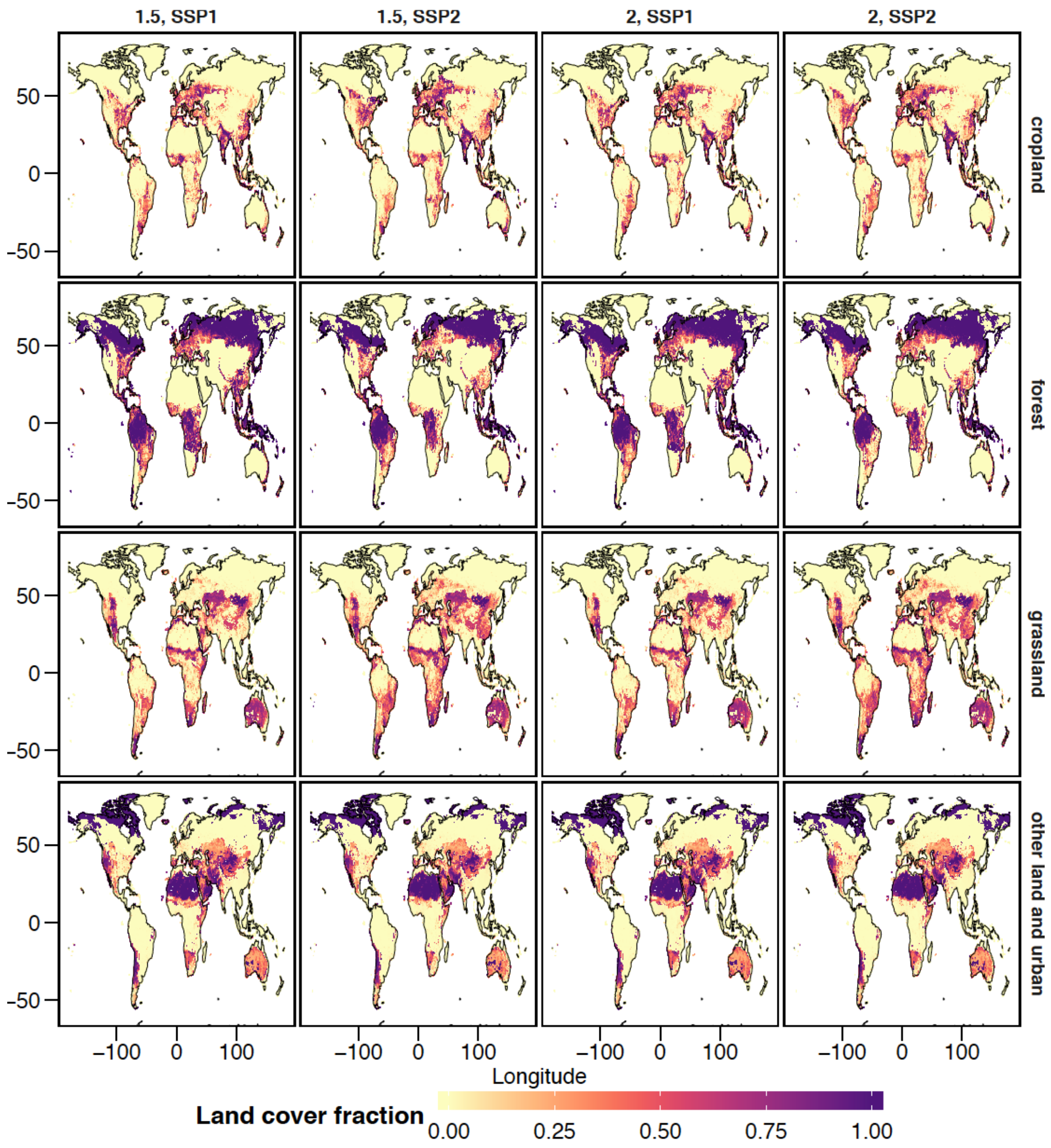

Supplementary Figure S17: Fraction of respective land cover classes in 2100 in IMAGE.

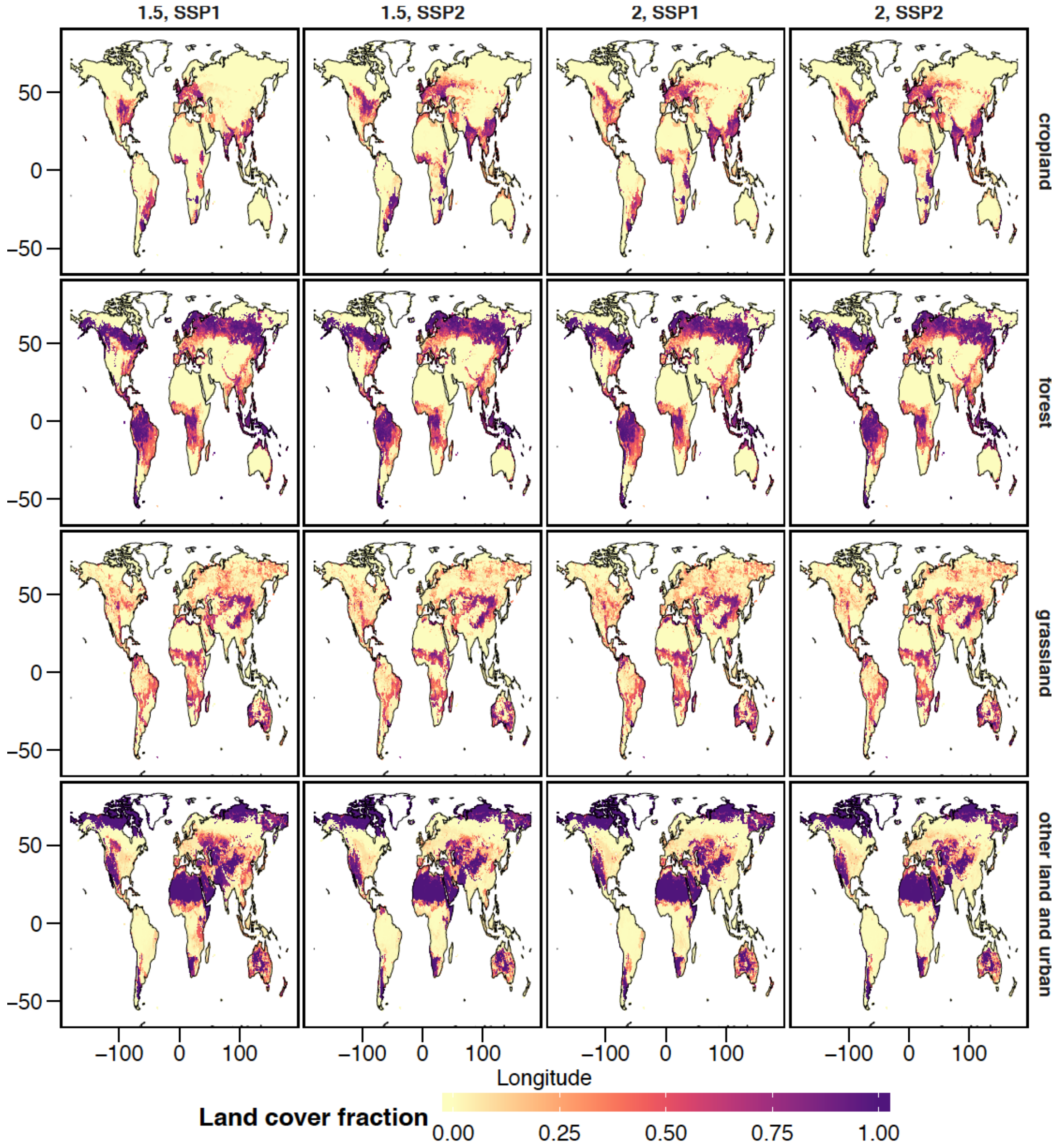

Supplementary Figure S18: Fraction of respective land cover classes in 2100 in MagPIE.

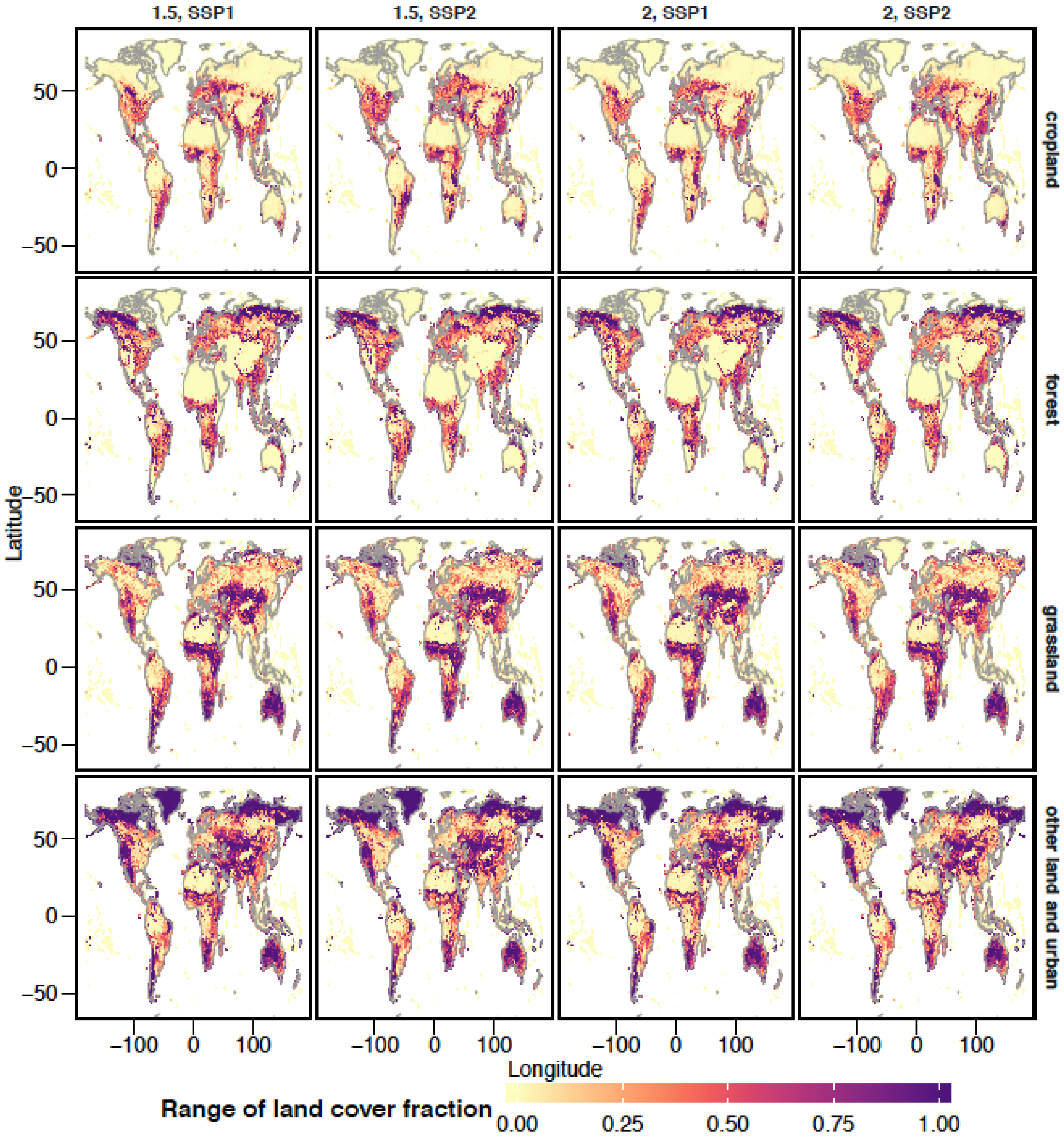

Supplementary Figure S19: Range of land cover fractions (ensemble maximum – ensemble minimum) among the analyzed IAMs in 2100 grouped by land cover type. The values are displayed as differences in fractional areas (unitless).

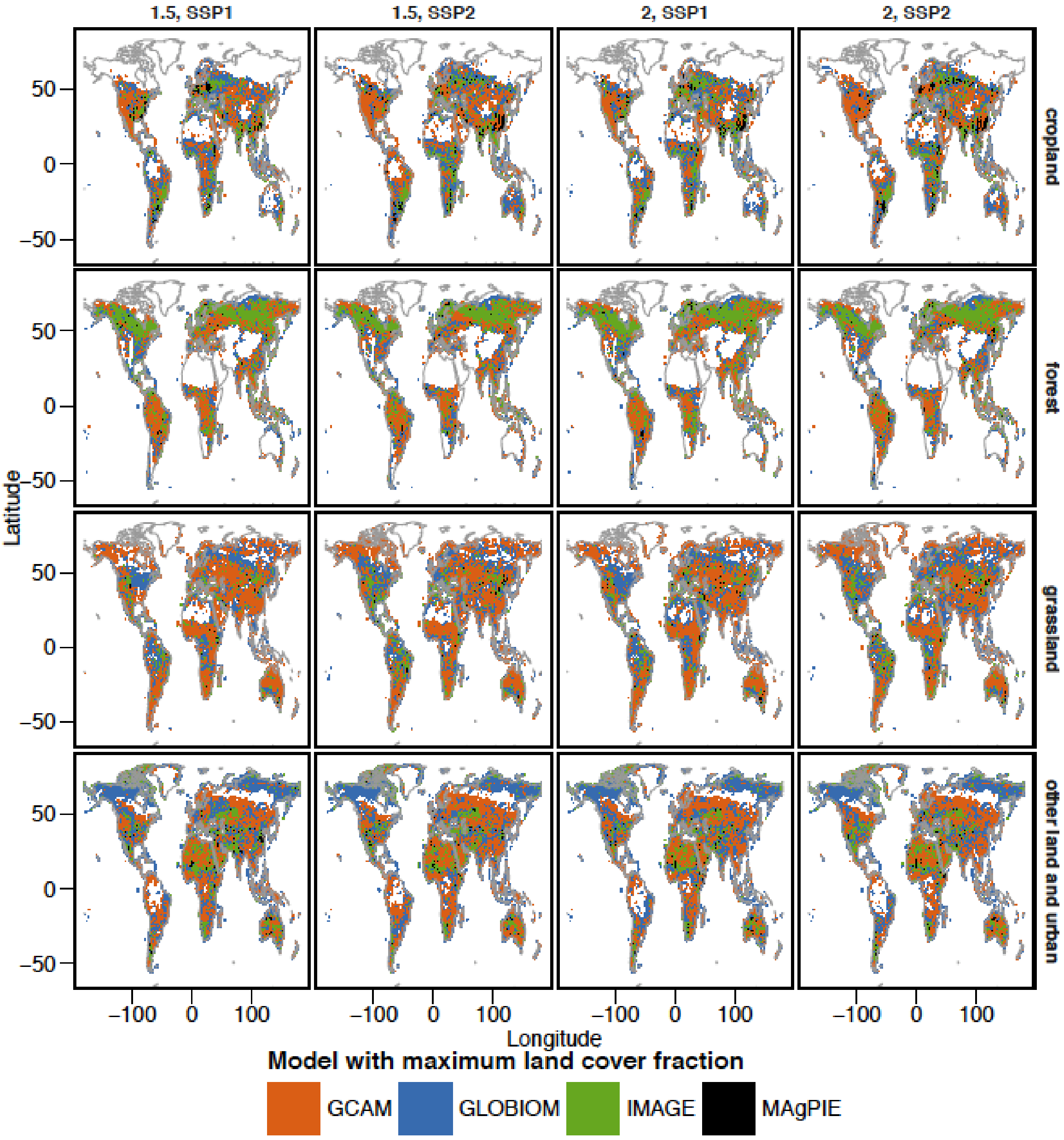

Supplementary Figure S20: Name of IAM model with maximum land cover fraction in 2100. Grid cells are masked if (a) the land cover fraction is less than or equal to 5%, or (b) there are less than two models with valid data.

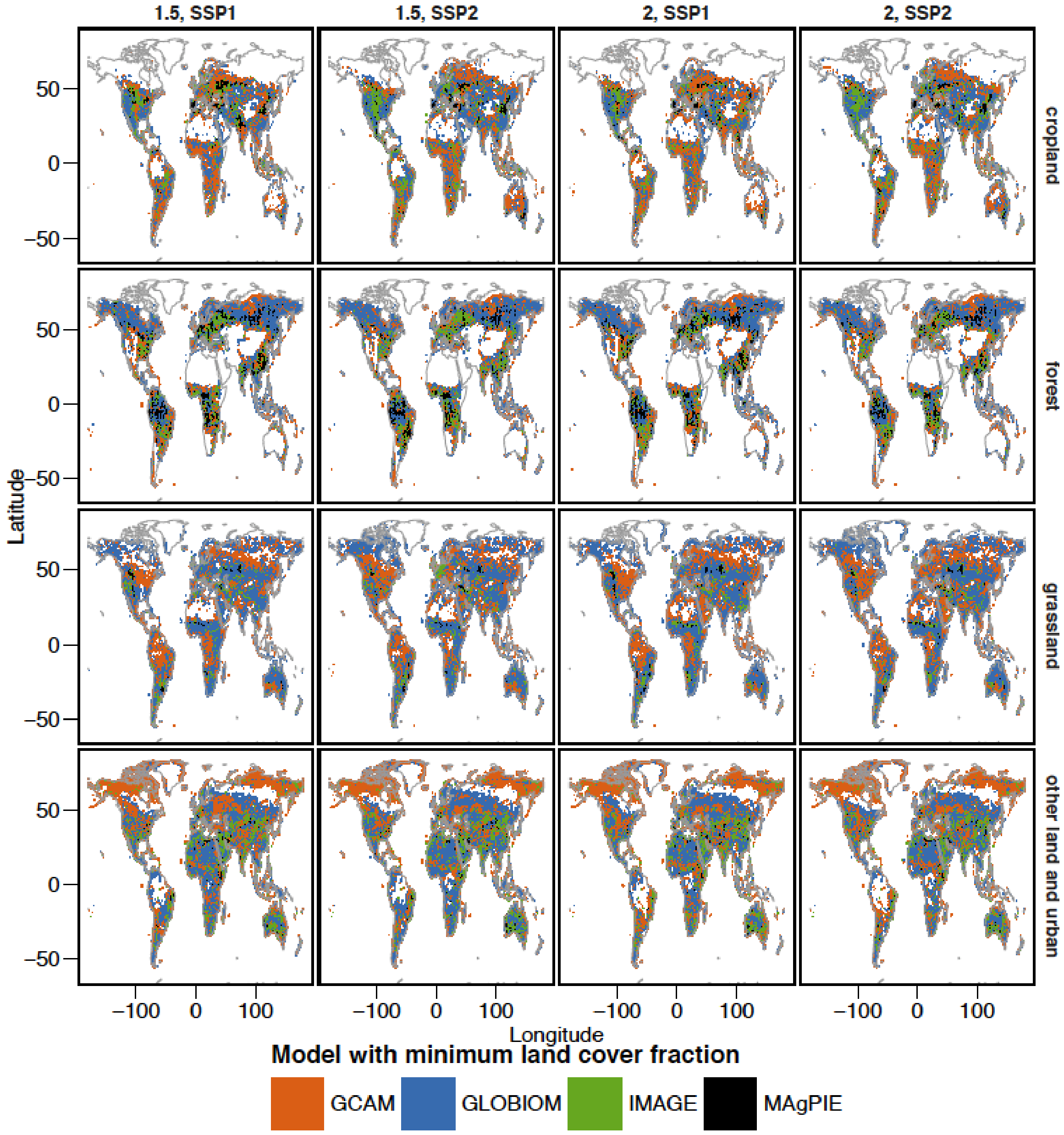

Supplementary Figure S21: Name of IAM model with minimum land cover fraction in 2100. Grid cells are masked if (a) the land cover fraction is less than or equal to 5%, or (b) there are less than two models with valid data.
